# The Phase Space Model of Nonrelativistic Quantum Mechanics

**DOI:** 10.3390/e23050581

**Published:** 2021-05-08

**Authors:** Jaromir Tosiek, Maciej Przanowski

**Affiliations:** Institute of Physics, Łódź University of Technology, 90-924 Łódź, Poland; maciej.przanowski@p.lodz.pl

**Keywords:** phase space quantum mechanics, Wigner function, discrete phase space

## Abstract

We focus on several questions arising during the modelling of quantum systems on a phase space. First, we discuss the choice of phase space and its structure. We include an interesting case of discrete phase space. Then, we introduce the respective algebras of functions containing quantum observables. We also consider the possibility of performing strict calculations and indicate cases where only formal considerations can be performed. We analyse alternative realisations of strict and formal calculi, which are determined by different kernels. Finally, two classes of Wigner functions as representations of states are investigated.

## 1. Introduction

In the twenties of the twentieth century, the young, brave scientists Werner Heisenberg and Erwin Schrödinger noticed that ‘old’ quantum theory had reached its end. To make real progress, it was necessary to propose a completely new formalism. Heisenberg, together with Born and Jordan [1,2,3] developed matrix calculus. A few months later, Schrödinger presented a wave version of quantum theory [4] and proved its equivalence [5] using the Heisenberg matrix calculus. Quantum mechanics gained its own mathematical language that was radically different from classical physics, and which has been successfully used to the present day.

On the other hand, the classical world is a limit of the quantum one. Therefore, it seems to be natural that an alternative to the Hilbert space version of quantum mechanics, compatible with classical physics, should exist. A promising candidate is the phase space formulation of quantum mechanics. The foundations of this approach have been given in the outstanding publications [6,7,8,9,10]. Several results illustrating the potential of quantum phase space physics were also achieved [11,12,13,14,15,16,17,18].

A new impulse in the development of phase space quantum mechanics took place in the 1970s, when F. Bayen, M. Flato, C. Fronsdal, A. Lichnerowicz, and D. Sternheimer presented an extended version of phase space calculus [19,20,21].

From that time, phase space quantum mechanics has remained present in the scientific world. It has developed in parallel as a part of physics and independently as a subdiscipline of mathematics called deformation quantisation. Among the books and review papers devoted to the topic, we recommend, e.g., [22,23,24,25,26,27,28,29,30].

Our review is focused on certain aspects of the phase space version of quantum physics. First, we discuss a structure of quantum phase space. We analyse which elements are required in the best known case of symplectic space $R^{2n},$ what else is demanding on other types of symplectic manifolds, and finally how to build discrete phase spaces.

Analogous considerations are made for observables. The main task is the construction of a noncommutative but associative ∗-product representing the multiplication of operators acting in a Hilbert space. The form of the ∗-product depends on the phase space. In the case of a symplectic manifold $R^{2n}$ or on a discrete phase space, a strict star calculus is known. However, in other situations, we have to deal with formal series.

The question closely related to the ∗-multiplication is a choice of ordering. Since quantum theory is more general than the classical theory, a fixed classical observable can be the classical counterpart of several quantum observables. Every quantum observable from that class is related to its classical limit by a relation called ordering. This statement implies that there exist several admissible ∗-products related to different orderings. We analyse physically acceptable orderings and show how they affect the ∗-products.

The last aspect of phase space quantum mechanics investigated in the current article is the representation of states. We propose two realisations of this task. Thus, we introduce a Wigner function as a counterpart of the density operator, and we study its fundamental properties and dependence on the choice of ordering. We discuss its functional action on observables and provide a time evolution formula for the Wigner function. In the case of discrete phase space, we construct a Wigner function in an alternative manner with the use of the trace of a density operator multiplied by a generalised Stratonovich–Weyl quantiser.

## 2. Some Comments on the Hilbert Space Formulation of Quantum Theory

One of the most significant features of physics is the quantitative description of natural phenomena. Over centuries, a universal scheme for the practical realisation of this goal has been determined. It consists of four elements. A basis for the model is some space on which we describe physical processes. This space is a set of objects usually equipped with some additional structures, such as a topology, a norm, or a metric. Then, we introduce on this set some elements representing measurable quantities called observables and elements determining states. To represent the results of measurements, we establish a mapping from pairs {observable, state} into real numbers.

The transformations of a physical system, especially a time evolution, are represented by mappings acting in a set of observables or in a collection of states.

Since our object of interest is quantum mechanics, we will sketch the implementation of the aforementioned postulates in that discipline. The reader who is more interested in the topic is encouraged to read, e.g., [31] or [32].

The currently used realisation of the mathematical model of quantum mechanics was proposed by Dirac [33,34] and von Neumann [35]. A modern rigorous version can be seen, e.g., in [36] or [37]. Let us state the main facts about this attempt.

According to the mentioned realisation of quantum theory, the stage is a separable Hilbert space H, which is further extended to a rigged Hilbert space. Since H is by definition a unitary space, it is equipped in a natural way with a norm, angles between vectors, a metric, and a topology. Its topological basis is countable. Since, by definition, a Hilbert space is complete, every Cauchy sequence in H is convergent.

The dimension of the separable Hilbert space can be finite or infinite. According to the Riesz–Fisher theorem, every separable Hilbert space of infinite dimension is isomorphic to the Hilbert space of the square summable series $l^{2}$, and every finite dimensional Hilbert space of dimension dimH=n is isomorphic to $C^{n}.$ Taking into account how different physical systems are modelled on isomorphic vector spaces, we can see that information about a certain physical system contained in its Hilbert space is very limited.

On the other hand, one can emphasise certain features of a set under consideration by choice of the realisation of the Hilbert space, such as the space of square integrable functions $L^{2}\left(R\right)$ on a 1-D axis or $L^{2}\left(R^{3}\right)$ on a 3-D volume.

For systems in which two or more disjoint features are modelled, e.g., a spin and a spatial motion, the total Hilbert space is represented by the tensor product of the respective Hilbert spaces

$$H=H_{\mathrm{spin}}⊗H_{\mathrm{space}}.$$

However, these endeavours do not enrich the information contained in space H as such.

Let us make a brief review of the assumptions about measurable quantities. Observables are represented by self- adjoint linear operators defined on some dense subspaces of H. An extended discussion of this postulate can be found in [38]. The eigenvalues of quantum observables are real, and their eigenvectors constitute bases of H; therefore, by performing a series of measurements related to quantities and performing a complete set of observables, we gain the maximal information about a state. Thus, the characteristics of the system under consideration are given by observables rather than by the Hilbert space.

Among all linear operators acting in H, we chose a special class of of bounded linear operators B(H) defined on the whole Hilbert space. This set constitutes a noncommutative ∗-A algebra with unity. The involution ’∗’ is implemented by operation of the Hermitian conjugate. Set B(H) contains operators representing measurable quantities. Algebra B(H) is also a Lie algebra with the Lie bracket being the commutator of operators

(1) $$\left[\hat{A},\hat{B}\right]=\hat{A}\hat{B}-\hat{B}\hat{A}.$$

In the third step—namely the construction of states—we perform following [39]. The starting point is the ∗-A algebra from the preceding paragraph.

By a quantum state, we mean every linear positive functional *f* over algebra ∗-A satisfying the normalisation condition, i.e., a functional, for which the following relations hold

(2) $$\forall \;\hat{A}\;,\;\hat{B}\in B\left(H\right)\;\;\;\forall \;a\;,\;b\in C\;\;\;f\left(a\hat{A}+b\hat{B}\right)=af\left(\hat{A}\right)+bf\left(\hat{B}\right),$$

(3) $$\forall \;\hat{A}\in B\left(H\right)\;\;\;f\left({\hat{A}}^{†}\hat{A}\right)\geq 0$$

and

(4) $$f(\hat{1})=1.$$

A practical realisation of this functional action is given by the trace of product of the given operator $\hat{A}$ with a special operator $\hat{\varrho }$ known as a density operator.

(5) $$f\left(\hat{A}\right):=\mathrm{Tr}\left(\hat{\varrho }\hat{A}\right),\;\;\;\hat{A}\in B\left(H\right),$$

A detailed analysis of properties of the density operator $\hat{\varrho }$ is presented in Section 6. The physical meaning of functional action (5) is revealed in a formula determining the average value of quantity $\hat{A}$

$$\langle \hat{A}\rangle :=\mathrm{Tr}\left(\hat{\varrho }\hat{A}\right).$$

To make our view panoramic, we remember that transformations, especially the time evolution, are represented by unitary operators according to the rule

(6) $${\hat{A}}^{\prime }=\hat{U}\hat{A}{\hat{U}}^{-1}.$$

The Hilbert space formulation of quantum theory is widely accepted, and we will refer to it throughout the paper. It works for simple systems. The fundamental reason seems to be the versatility of the Hilbert spaces on which we model the systems. In classical physics, the phase space of a particle moving on a sphere and of a free particle are different. In quantum mechanics, these two cases are represented on the same (up to an isomorphism) Hilbert space. Currently, we have, at our disposal, a method of introducing Cartesian position operators and conjugated momenta operators; however, there is no method of building analogous operators in curvilinear coordinates. Overcoming this serious obstacle is necessary to quantise systems with constraints or with curved phase spaces.

## 3. The Structure of Quantum Phase Space

In contrast to the Hilbert space approach to quantum physics, in classical mechanics, we deal with constraints or with curvature without problems and the mathematical apparatus of differential geometry used for that purpose is well developed. Since classical physics is a limit of quantum mechanics, it is natural that a mathematical model common for these two theories should exist, and that, in the frame of this calculus, we would be able to analyse quantum effects with constraints or with complicated geometry. In this section, we begin construction of that description.

Following the observations from the previous paragraph, we first consider systems that are classically modelled on phase space $R^{2}.$ The generalisation of the presented results on phase space $R^{2n}$ is straightforward.

The original problem, stated and partially solved by Dirac, was to propose a procedure of assigning linear operators acting in the Hilbert space H and representing measurable quantities to some functions on phase space $R^{2}.$ As it is well known, this procedure, called quantisation, consists of two steps. In the first step, we propose self-adjoint operators of position $\hat{q}$ and momentum $\hat{p}.$ It is required that they satisfy the commutation relation

(7) $$\left[\hat{q},\hat{p}\right]=i\hbar \hat{1}.$$

Having operators of position and momentum, we can build an algorithm enabling us to find an operator on the Hilbert space H representing the function A(p,q) on the classical phase space. This mapping is called the generalised Weyl application [40] and is expressed by the formula

(8) $$\hat{A}=W_{P}\left(A\left(p,q\right)\right)=\frac{1}{{\left(2\pi \right)}^{2}}\int_{R^{2}}\tilde{A}\left(\lambda ,\mu \right)P\left(\hbar \lambda \mu \right)\exp\left[i\left(\lambda \hat{p}+\mu \hat{q}\right)\right]d\lambda d\mu ,$$

where P(ℏλμ) is some function and

$$\tilde{A}\left(\lambda ,\mu \right):=\int_{R^{2}}A\left(p,q\right)\exp\left[-i\left(\lambda p+\mu q\right)\right]dpdq$$

is the Fourier transform of the function A(p,q). It is required for function A(p,q) to have the Fourier transform, which reduces the class of objects to which the generalised Weyl application can be applied. However, in practical physical problems, measurable quantities represented by rapidly growing functions of *p* and *q* appear rarely.

By direct calculation, one can see that, e.g.,

$$\forall \;n\in N\;W_{P}\left(p^{n}\right)=P\left(0\right){\hat{p}}^{n}\;\;\;,\;\;\;W_{P}\left(q^{n}\right)=P\left(0\right){\hat{q}}^{n}$$

but

$$W_{P}\left(p\cdot q\right)=P\left(0\right)\hat{q}\hat{p}-\frac{\hbar }{2}P^{\prime }\left(0\right)-\frac{i\hbar }{2}P\left(0\right).$$

One usually assumes that classically measurable quantities called observables are represented by smooth real functions on the phase space (as in quantum mechanics). Earlier, we said that quantum observables are implemented by self-adjoint operators; to ensure that real functions turn in self-adjoint operators via the Weyl application, we need to require the function P(ℏλμ) to be real. A possible choice of that function, equivalent to the selection of operator ordering, will be discussed later in Section 5.

In the literature (see [41,42]), formula (8) is often written with the use of the generalised Stratonovich–Weyl quantiser or the Fano operators also called the generalised Grossmann–Royer operator ${\hat{\Phi }}_{P}\left(p,q\right)$ as

(9) $$W_{P}\left(A\left(p,q\right)\right)=\frac{1}{2\pi \hbar }\int_{R^{2}}A\left(p,q\right)\hat{\Phi }\left[P\right]\left(p,q\right)dpdq,$$

where $\hat{\Phi }\left[P\right]\left(p,q\right)$ is an operator valued function on plane $R^{2}$ of the form

(10) $$\hat{\Phi }\left[P\right]\left(p,q\right):=\frac{\hbar }{2\pi }\int_{R^{2}}P\left(\hbar \lambda \mu \right)\exp\left\{i\lambda \left(\hat{p}-p\right)+i\mu \left(\hat{q}-q\right)\right\}d\lambda d\mu .$$

On a 2n-dimensional space, $R^{2n}$ relations (9) and (10) transform into

(11) $$W_{P}\left(A\left(p_{1},\ldots ,q^{n}\right)\right)=\frac{1}{{\left(2\pi \hbar \right)}^{n}}\int_{R^{2n}}A\left(p_{1},\ldots ,q^{n}\right)\hat{\Phi }\left[P\right]\left(p_{1},\ldots ,q^{n}\right)dp_{1}\cdots dq^{n},$$

with the generalised Stratonovich–Weyl quantiser equal to

$$\hat{\Phi }\left[P\right]\left(p_{1},\ldots ,q^{n}\right):=\frac{\hbar^{n}}{{\left(2\pi \right)}^{n}}\int_{R^{2n}}P\left(\hbar \sum_{j=1}^{n}\lambda^{j}\mu_{j}\right)$$

(12) $$\times \exp\left\{i\sum_{j=1}^{n}\lambda^{j}\left({\hat{p}}_{j}-p_{j}\right)+i\sum_{j=1}^{n}\mu_{j}\left({\hat{q}}^{j}-q^{j}\right)\right\}d\lambda^{1}\cdots d\mu_{n}.$$

The shape of argument of function P will be explained in Section 5.

An observation crucial for our purposes is that in the case when a classical counterpart of our quantum system is modelled on the phase space $R^{2n},$ one can use a transformation inverse to the generalised Weyl application to construct a phase space image of quantum mechanics. One of the equivalent forms of this transformation known as the generalised Weyl correspondence for n=1 is given by the expression

$$W_{P}^{-1}\left(\hat{A}\right)\left(p,q\right)=\frac{1}{{\left(2\pi \right)}^{2}}\int_{R^{4}}dp^{\prime }dq^{\prime }d\lambda d\mu P^{-1}\left(\hbar \lambda \mu \right)$$

(13) $$\times \exp\left(-\frac{i\hbar \lambda \mu }{2}\right)\exp\left[i\lambda \left(p-p^{\prime }\right)+i\mu \left(q-q^{\prime }\right)\right]\frac{\langle q^{\prime }\left|\hat{A}\right|p^{\prime }\rangle }{\langle q^{\prime }|p^{\prime }\rangle }.$$

From formula (13), we can deduce that, indeed, the space on which we build quantum theory is $R^{2}.$ By $|p^{\prime }\rangle ,|q^{\prime }\rangle$, we mean eigenvectors of operators of momentum $\hat{p}$ and position $\hat{q}$ referring to the eigenvalues $p^{\prime },\;q^{\prime }$, respectively. The operators $\hat{p},\;\hat{q}$ are taken in Cartesian coordinates so that they satisfy the commutation rule (7).

As one can check easily, e.g.,

$$\forall \;n\in N\;W_{P}^{-1}\left({\hat{p}}^{n}\right)=P^{-1}\left(0\right)p^{n}\;\;\;,\;\;\;W_{P}^{-1}\left({\hat{q}}^{n}\right)=P^{-1}\left(0\right)q^{n}$$

but

$$W_{P}^{-1}\left(\hat{q}\hat{p}\right)=P^{-1}\left(0\right)pq+\frac{i\hbar }{2}P^{-1}\left(0\right)-\frac{\hbar }{2}{P^{-1}}^{\prime }\left(0\right).$$

By construction of the generalised Weyl correspondence, we can see that the space $R^{2},$ on which an alternative approach to quantum world is proposed, has to be classical phase space $R^{2}.$ Thus, this space is a symplectic manifold and, in canonical coordinates (p,q), related with the operators $\hat{p},\;\hat{q}$. By the generalised Weyl correspondence, the symplectic form is represented by expression

ω=dp∧dq.

The existence of the symplectic structure enables us to calculate the volume of any domain of a symplectic manifold. As a differentiable manifold, the phase space is a topological space. It can be covered with one chart, in which the global coordinates are *p* and *q*. $R^{2}$ is also a vector space; however, this fact plays a minor role.

Let us make some additional comments about formula (13). These remarks will be useful in the context of systems represented in finite dimensional Hilbert spaces.

Generalised eigenvectors of self-adjoint unbounded operators $\hat{p},\;\hat{q}$ constitute two bases of H: ${\left\{|p\rangle \right\}}_{p=-\infty }^{\infty }$ and ${\left\{|q\rangle \right\}}_{q=-\infty }^{\infty }$, respectively. These bases are orthonormal

$$\langle p^{\prime }|p\rangle =\delta \left(p-p^{\prime }\right)\;\;\;,\;\;\;\langle q^{\prime }|q\rangle =\delta \left(q-q^{\prime }\right).$$

The reader interested in the mathematically strict formulation of the aforesaid properties is encouraged to look into, e.g., [38].

The decompositions of the operators of momentum and position in the bases constituted by their eigenvectors lead to the formulas

(14) $$\hat{p}=\int_{R}p\left|p\rangle \langle p|dp\;\;\;\mathrm{and}\;\;\;\hat{q}=\int_{R}q|q\rangle \langle q\right|dq.$$

Starting from the operators $\hat{p}$ and $\hat{q}$, we introduce two families of unitary operators:

(15) $$\exp\left(i\lambda \hat{p}\right)\;\;\;\mathrm{and}\;\;\;\exp\left(i\mu \hat{q}\right),\;\;\lambda ,\mu \in R$$

where λ,μ∈R. These operators appear in the generalised Weyl application (8) and satisfy the following commutation rule

$$\exp\left(-\frac{i\hbar \lambda \mu }{2}\right)\exp\left(i\lambda \hat{p}\right)\exp\left(i\mu \hat{q}\right)=\exp\left(\frac{i\hbar \lambda \mu }{2}\right)\exp\left(i\mu \hat{q}\right)\exp\left(i\lambda \hat{p}\right)$$

(16) $$=\exp\left\{i\left(\lambda \hat{p}+\mu \hat{q}\right)\right\}=:\hat{U}\left(\lambda ,\mu \right).$$

With the use of the operators $\hat{U}\left(\lambda ,\mu \right)$, the Weyl correspondence turns into

(17) $$W_{P}^{-1}\left(\hat{A}\right)\left(p,q\right)=\frac{\hbar }{2\pi }\int_{R^{2}}d\lambda d\mu \;P^{-1}\left(\hbar \lambda \mu \right)\exp\left\{i\left(\lambda p+\mu q\right)\right\}\mathrm{Tr}\left\{\hat{A}\;{\hat{U}}^{†}\left(\lambda ,\mu \right)\right\}.$$

Now, we extend our former results on systems that are classically are modelled on symplectic manifolds that are different from $R^{2n}.$ Among them are systems with constraints and objects living in curved configuration spaces. The fundamental difference between these systems and the ones discussed before is a local character of the coordinates *p* and *q* Therefore, we cannot use the Fourier transform and, thus, our formulas for the generalised Weyl application (8) as well as for the generalised Weyl correspondence (13) are useless. For discussion on the problem with the Stratonovich–Weyl quantiser for an arbitrary Riemannian manifold see [43] and the references therein.

Taking into account our earlier remarks about the universality of the Hilbert space H, we assume however, that a Hilbert space representation of these systems may be built on H.

Since we predict that there exists a mapping

SW:A(M)→BB(H)

between a linear space of functions A(M) on a phase space M and some subspace, say BB(H), of the vector space of all linear operators acting in the Hilbert space H, we can impose some natural restrictions on the mapping SW known as the generalised Stratonovich–Weyl application.

Commonly accepted conditions implemented in this mapping are the following:

1. Mapping SW establishes a one-to-one correspondence between elements of the two linear spaces A(M) and BB(H). Since we do not know whether manifold M is the phase space of the classical counterpart of our system, this demand offers an opportunity to investigate a structure of space M.
  To achieve this goal, we introduce an inverse operation
  $$SW^{-1}:BB\left(H\right)\to A\left(M\right)$$
  known as the generalised Stratonovich–Weyl correspondence. At this moment, we do not discuss the problem of whether the mappings $SW,\;SW^{-1}$ establish an isomorphism between the potential algebras A(M) of functions and BB(H) of operators, which will be built on the linear spaces A(M) and BB(H).
  Since, for our purposes, the generalised Stratonovich–Weyl correspondence is more useful, we will present the next assumptions for it.
2. Mapping $SW^{-1}$ is C-linear.
3. $SW^{-1}\left(\hat{1}\right)=1$, i.e., the constant function equal to 1 on the whole phase space is a counterpart of the identity operator acting in the Hilbert space H.
4. The image of a self-adjoint operator in the generalised Stratonovich–Weyl correspondence is a real function. We extend this requirement to the form
  $$SW^{-1}\left({\hat{A}}^{†}\right)=\bar{SW^{-1}\left(\hat{A}\right)}.$$

Unfortunately, requirements 1–4 are not sufficient to reveal the structure of the phase space M, on which functions belonging to the set A(M) are defined. Hence, we are not able to prove that the classical phase space is suitable for the quantised set. Thus, we usually assume the classical phase space to be also the quantum one. We are also aware of exceptions, like sets whose classical phase space is a cylinder $S^{1}\times R.$ A detailed analysis of this situation can be found in [44,45,46,47].

At the end of this section, we will discuss a phase space representation of quantum systems modelled on finite dimensional Hilbert spaces $H^{\left(s+1\right)}$ where $s+1=\dim H^{\left(s+1\right)}<\infty .$ This part is based on our previous papers [48,49] (see also the list of references therein). The idea of the construction of phase space for sets of this kind is an adaptation of a scheme arising from the generalised Weyl correspondence on $R^{2n}$.

First, we choose a a basis

(18) $$\{|0\rangle ,|1\rangle ,\ldots ,|s\rangle \},\;\;\langle n|n^{\prime }\rangle =\delta_{nn^{\prime }},\;\;n,n^{\prime }=0,1,\ldots ,s$$

in $H^{\left(s+1\right)}.$ An alternative system of vectors spanning this Hilbert space is

(19) $$\begin{array}{c} |\phi_{m}\rangle :=\frac{1}{\sqrt{s+1}}\sum_{n=0}^{s}\exp\left(in\phi_{m}\right)|n\rangle ,\\ \langle \phi_{m}|\phi_{m^{\prime }}\rangle =\delta_{mm^{\prime }}\;\;,\;\;m,m^{\prime }=0,1,\ldots ,s. \end{array}$$

Real numbers $\phi_{m}$ enumerating versors are defined as

$$\phi_{m}=\phi_{0}+\frac{2\pi }{s+1}m,\;\;m=0,1,\ldots ,s,\;\;\phi_{0}\in R.$$

One can put $\phi_{0}=0$, and we do that.

Let us introduce two self-adjoint operators known as the Schwinger operators, whose eigenvalues are numbers *n* and $\phi_{m}.$ Projective operators $\left|n\rangle \langle n|,|\phi_{m}\rangle \langle \phi_{m}\right|$ project vectors from $H^{\left(s+1\right)}$ on the eigenstates of these two operators.

(20) $$\hat{n}:=\sum_{n=0}^{s}n|n\rangle \langle n|\;\;,\;\;\hat{\phi }:=\sum_{m=0}^{s}\phi_{m}|\phi_{m}\rangle \langle \phi_{m}|.$$

Operators $\hat{n}$ and $\hat{\phi }$ do not commute because

$$\left[\hat{n},\hat{\phi }\right]=\frac{2\pi }{s+1}\sum_{l=0}^{s}\sum_{\begin{array}{c} m=0\\ m\neq l \end{array}}^{s}\frac{l-m}{\exp\left(\frac{2i(l-m)\pi }{s+1}\right)-1}|\phi_{m}\rangle \langle \phi_{l}|.$$

Having those operators, we introduce two families of the unitary operators:

(21) $$\hat{V}:=\exp\left(i\frac{2\pi }{s+1}\hat{n}\right)\;\;\mathrm{and}\;\;\hat{U}:=\exp\left(i\hat{\phi }\right)$$

fulfilling the conditions ${\hat{V}}^{s+1}=\hat{1}$ and ${\hat{U}}^{s+1}=\exp\left\{i\left(s+1\right)\phi_{0}\right\}\hat{1}$, respectively.

These operators satisfy the commutation relation analogous to the operators $\exp(i\lambda \hat{p})$ and $\exp(i\mu \hat{q})$, i.e.,

(22) $$\exp\left(-i\frac{\pi kl}{s+1}\right){\hat{U}}^{k}{\hat{V}}^{l}=\exp\left(i\frac{\pi kl}{s+1}\right){\hat{V}}^{l}{\hat{U}}^{k}=:\hat{D}\left(k,l\right),\;\;k,l\in Z.$$

Therefore, we can propose an explicit correspondence between some operator $\hat{A}$ acting in the Hilbert space $H^{\left(s+1\right)}$ and a function $A(\phi_{m},n)$ of two discrete real arguments $\phi_{m}$ and n.

(23) $$A\left(\phi_{m},n\right)=\frac{1}{s+1}\sum_{k,l=0}^{s}K^{-1}\left(k,l\right)\exp\left\{i\left(k\phi_{m}+\frac{2\pi }{s+1}ln\right)\right\}\times \mathrm{Tr}\left\{\hat{A}\;{\hat{D}}^{†}\left(k,l\right)\right\}.$$

Function $A(\phi_{m},n)$ is defined on a discrete phase space (a grid) ${\left\{(\phi_{m},n)\right\}}_{m,n=0}^{s}$ denoted by $\Gamma^{\left(s+1\right)}.$ Therefore, we see that a phase space counterpart of $H^{\left(s+1\right)}$ is a lattice $\Gamma^{\left(s+1\right)}.$ The grid, of course, is not a differentiable manifold.

Kernel K(k,l), like the function P(ℏλμ), is responsible for the choice of ordering. Th question of physically acceptable orderings will be discussed later.

As an example, we consider the case when s=1. Thus, bases of the Hilbert space $H^{\left(2\right)}$ are: $\{|0\rangle ,|1\rangle \}$ and alternatively $\{|\phi_{0}\rangle =\frac{1}{\sqrt{2}}\left(|0\rangle +|1\rangle \right),|\phi_{1}\rangle =\frac{1}{\sqrt{2}}\left(|0\rangle -|1\rangle \right)\}.$ The fundamental operators $\hat{n}$ and $\hat{\phi }$ are equal to

$$\hat{n}=|1\rangle \langle 1|\;\;\;,\;\;\;\hat{\phi }=\pi |\phi_{1}\rangle \langle \phi_{1}|.$$

According to the rule (23), the function representing operator $\hat{n}$ equals

$$n\left(\phi_{m},n\right)=\left[\begin{array}{cc} n(\phi_{0},0) & n(\phi_{0},1)\\ n(\phi_{1},0) & n(\phi_{1},1) \end{array}\right]=\frac{1}{2}\left[\begin{array}{cc} K^{-1}\left(0,0\right)-K^{-1}\left(0,1\right) & K^{-1}\left(0,0\right)+K^{-1}\left(0,1\right)\\ K^{-1}\left(0,0\right)-K^{-1}\left(0,1\right) & K^{-1}\left(0,0\right)+K^{-1}\left(0,1\right) \end{array}\right].$$

Exactly as in the case of the phase space $R^{2n}$, a relation between some functions on grid $\Gamma^{\left(s+1\right)}$ and a class of linear operators acting on the Hilbert space $H^{\left(s+1\right)}$ is a bijection. Thus, the inverse formula to (23) exists and is of the form

(24) $$\hat{A}=\frac{1}{s+1}\sum_{m,n=0}^{s}A\left(\phi_{m},n\right)\hat{\Omega }\left[K\right]\left(\phi_{m},n\right),$$

where the Stratonovich–Weyl quantiser equals

(25) $$\hat{\Omega }\left[K\right]\left(\phi_{m},n\right):=\frac{1}{s+1}\sum_{k,l=0}^{s}K\left(k,l\right)\exp\left\{-i\left(k\phi_{m}+\frac{2\pi }{s+1}ln\right)\right\}\hat{D}\left(k,l\right).$$

For objects that are modelled on the Hilbert space $H⊗H^{\left(s+1\right)}$ and for which the generalised Weyl correspondence works, the respective phase space is the Cartesian product $R^{2n}\times \Gamma^{\left(s+1\right)}.$ As it is easy to check, the bijection between some class of linear operators and functions on $R^{2}\times \Gamma^{\left(s+1\right)}$ is given by the pair of relations

(26) $$\begin{array}{c} A\left(p,q,\phi_{m},n\right)=\frac{\hbar }{2\pi }\frac{1}{s+1}\sum_{k,l=0}^{s}\int_{R^{2}}d\lambda d\mu {\left(P\left(\hbar \lambda \mu \right)K\left(kl\right)\right)}^{-1}\\ \;\;\;\;\;\;\;\;\;\;\;\;\;\;\;\;\;\;\;\;\;\;\;\;\;\times \exp\left\{i\left(\lambda p+\mu q\right)\right\}\exp\left\{i\frac{2\pi }{s+1}\left(km+ln\right)\right\}\mathrm{Tr}\left\{\hat{A}\;{\hat{U}}^{†}\left(\lambda ,\mu \right){\hat{D}}^{†}\left(k,l\right)\right\} \end{array}$$

and

$$\hat{A}=\frac{1}{{\left(2\pi \right)}^{2}{\left(s+1\right)}^{2}}\sum_{k,l,m,n=0}^{s}\int_{R^{4}}d\lambda d\mu \;dpdq\;P\left(\hbar \lambda \mu \right)K\left(kl\right)\times$$

(27) $$\exp\left\{-i\left(\lambda p+\mu q\right)\right\}\exp\left\{-i\frac{2\pi }{s+1}\left(km+ln\right)\right\}A\left(p,q,\phi_{m},n\right)\hat{U}\left(\lambda ,\mu \right)\hat{D}\left(k,l\right).$$

For the motivation of putting $K\left(k,l\right)=K\left(kl\right)$ see Section 5. As one can see, the aforementioned bijection is based on the Fourier transform and the discrete Fourier transform. This is why we do not have a universal scheme to obtain the Hilbert space representation of a classical system modelled on some nontrivial symplectic space and why in having a Hilbert space representation of a nontrivial system, we may not be able to build its quantum phase space counterpart. It is possible that there exist different phase spaces on which the same quantum system is represented. An excellent illustration of this option is the description of a particle with spin [50,51].

## 4. Algebra of Quantum Observables

Thus far, to establish relations between a phase space and a Hilbert space, we explored the generalised Weyl correspondence/application and the generalised Stratonovich–Weyl mappings only at the level of linear structures of sets of observables. However, the total structures of the family of functions or family of operators used in quantum mechanics are much more extensive. The current section is devoted to an analysis of that topic.

As we already mentioned, classical observables are smooth, real functions on the phase space M of a system. Their multiplication is the usual pointwise product of functions. Thus, observables are real elements of the ring of smooth, complex, valued functions $(C^{\infty }\left(M\right),+,\cdot ),$ which is an algebra over the field of complex numbers C. Convergence in this algebra is introduced in agreement with the notion of convergence of generalised functions (see [52]). The sequence of functions ${\left\{A_{r}\left(p_{1},\ldots ,q^{n}\right)\right\}}_{r=1}^{\infty },\;\dim M=2n$ is convergent to a function $A_{0}\left(p_{1},\ldots ,q^{n}\right)$ if, on every compact subset of the manifold M, every sequence of partial derivatives ${\left\{\frac{\partial^{m_{1}+m_{2}+\ldots +m_{2n}}}{\partial^{m_{1}}p_{1}\ldots \partial^{m_{2n}}q^{n}}A_{r}\left(p_{1},\ldots ,q^{n}\right)\right\}}_{r=1}^{\infty }$ is uniformly convergent to the derivative $\frac{\partial^{m_{1}+m_{2}+\ldots +m_{2n}}}{\partial^{m_{1}}p_{1}\ldots \partial^{m_{2n}}q^{n}}A_{0}\left(p_{1},\ldots ,q^{n}\right).$ The definition may seem to be very restrictive but it well represents the physical meaning of convergence for measurable quantities. We present it in a local Darboux chart; however, of course it does not depend on the choice of coordinates.

The ring $\left(C^{\infty }\left(M\right),+,\cdot \right)$ in physics is equipped with a Lie algebra structure. This structure known as the Poisson bracket is introduced by the symplectic structure and locally in the Darboux chart

$${\left\{A\left(p_{1},\ldots ,q^{n}\right),B\left(p_{1},\ldots ,q^{n}\right)\right\}}_{P}$$

(28) $$=\sum_{l=1}^{n}\left(\frac{\partial A(p_{1},\ldots ,q^{n})}{\partial q^{l}}\frac{\partial B(p_{1},\ldots ,q^{n})}{\partial p_{l}}-\frac{\partial A(p_{1},\ldots ,q^{n})}{\partial p_{l}}\frac{\partial B(p_{1},\ldots ,q^{n})}{\partial q^{l}}\right).$$

Analysing the relationship between the Hilbert space formulation of quantum mechanics and its formulation on phase space $R^{2n}$, we propose some universal postulates referring to the more general case when the quantum phase space is a symplectic manifold M.

Observables are again represented by smooth real functions on the phase space M. The topology is as in the classical case. What is new, is the elements of the ring $(C^{\infty }\left(M\right),+,\cdot ),$ may depend on the Planck constant *ℏ*; although, in classical physics, this is not forbidden.

The fundamental difference between the sets of classical and of quantum observables lies in their products. Since the multiplication of linear operators acting on a Hilbert space H is associative but nonabelian, analogous properties are expected in the set of quantum observables on the phase space.

Therefore, we are forced to introduce a new product, represented by the symbol ∗ and called a ‘star’ product. Since its form depends on the structure of the manifold M, we need to, again, consider three options.

On a trivial phase space $R^{2}$, an explicit form of the ∗- multiplication can be found with the use of the generalised Weyl correspondence. As one proves

$$W_{P}^{-1}\left(\hat{A}\cdot \hat{B}\right)=W_{P}^{-1}\left(\hat{A}\right){*}_{P}W_{P}^{-1}\left(\hat{B}\right)\left(p,q\right)=A\left(p,q\right){*}_{P}B\left(p,q\right)$$

$$=\frac{1}{\hbar^{2}{\left(2\pi \right)}^{4}}\int_{R^{8}}d\lambda d\mu dp^{\prime }dq^{\prime }dp^{\prime \prime }dq^{\prime \prime }dp^{\prime \prime \prime }dq^{\prime \prime \prime }P^{-2}\left(\hbar \lambda \mu \right)\exp\left\{i\left[\lambda \left(p-p^{\prime }\right)+\mu \left(q-q^{\prime }\right)\right]\right\}$$

(29) $$\times A\left(p^{\prime \prime },q^{\prime \prime }\right)\mathrm{Tr}\left\{\hat{\Phi }\left[P\right]\left(p^{\prime },q^{\prime }\right)\;\hat{\Phi }\left[P\right]\left(p^{\prime \prime },q^{\prime \prime }\right)\;\hat{\Phi }\left[P\right]\left(p^{\prime \prime \prime },q^{\prime \prime \prime }\right)\right\}B\left(p^{\prime \prime \prime },q^{\prime \prime \prime }\right).$$

where $A\left(p,q\right)=W_{P}^{-1}\left(\hat{A}\right),\;B\left(p,q\right)=W_{P}^{-1}\left(\hat{B}\right).$ Equivalently ∗-multiplication of the functions A(p,q),B(p,q) is represented by the integral

(30) $$\left(A{*}_{P}B\right)\left(p,q\right)=\int_{R^{2}}d\lambda d\mu \;P^{-1}\left(\hbar \lambda \mu \right)\exp\left\{i\left(\lambda p+\mu q\right)\right\}\left(\tilde{\tilde{A}}⊠\tilde{\tilde{B}}\right)\left(\lambda ,\mu \right).$$

The ⊠-product does not depend on kernel P.

Auxiliary objects $\tilde{\tilde{A}}\left(\lambda ,\mu \right),\tilde{\tilde{B}}\left(\lambda ,\mu \right)$ are defined by

(31) $$\tilde{\tilde{A}}\left(\lambda ,\mu \right)=\frac{1}{{\left(2\pi \right)}^{2}}\;P\left(\hbar \lambda \mu \right)\int_{R^{2}}dpdq\exp\left\{-i\left(\lambda p+\mu q\right)\right\}A\left(p,q\right).$$

The ⊠-multiplication on phase space $R^{2}$ one is calculated as (compare [49])

$$\left(\tilde{\tilde{A}}⊠\tilde{\tilde{B}}\right)\left(\lambda ,\mu \right)=\int_{R^{4}}d\lambda^{\prime }d\mu^{\prime }d\lambda^{\prime \prime }d\mu^{\prime \prime }\tilde{\tilde{A}}\left(\lambda {\;}^{\prime },\mu {\;}^{\prime }\right)\exp\left\{\frac{i\hbar }{2}\left(\lambda {\;}^{\prime }\mu -\lambda \mu {\;}^{\prime }\right)\right\}$$

(32) $$\times \delta \left(\lambda {\;}^{\prime }+\lambda {\;}^{\prime \prime }-\lambda \right)\delta \left(\mu {\;}^{\prime }+\mu {\;}^{\prime \prime }-\mu \right)\tilde{\tilde{B}}\left(\lambda {\;}^{\prime \prime },\mu {\;}^{\prime \prime }\right).$$

Since the ${*}_{P}$-multiplication is based on the generalised Weyl correspondence, its explicit form is known only on the phase spaces $R^{2n}.$ The ∗-product is nonlocal.

We indicated that the set of bounded operators B(H) is also a Lie algebra. Therefore, a Lie algebra structure is mapped to the recently built algebra $(C^{\infty }\left(R^{2}\right),+,{*}_{P}).$ The Lie structure is called the Moyal bracket

(33) $${\left\{A\left(p,q\right),B\left(p,q\right)\right\}}_{M}:=\frac{1}{i\hbar }\left(A\left(p,q\right){*}_{P}B\left(p,q\right)-B\left(p,q\right){*}_{P}A\left(p,q\right)\right).$$

The Lie structure given by the Moyal bracket differs from the one determined by the Poisson bracket. Therefore, we can conclude that the classical Poisson algebra $\left(C^{\infty }\left(R^{2}\right),+,\cdot ,{\left\{\cdot ,\cdot \right\}}_{P}\right)$ of functions turns in the quantum case in a family of algebras $({\tilde{C}}^{\infty }\left(R^{2}\right),+,{*}_{P},{\left\{\cdot ,\cdot \right\}}_{M}).$ A possible presence of the Planck constant *ℏ* does not change the class of functions $C^{\infty }\left(R^{2}\right).$ However, being precise, not every two smooth functions from $C^{\infty }\left(R^{2}\right)$ can be multiplied in the sense of the star product ${*}_{P}.$ This complicated issue was discussed in [40,53]. Therefore, the vector space ${\tilde{C}}^{\infty }\left(R^{2}\right)$ is a subspace of $C^{\infty }\left(R^{2}\right).$

To introduce a ∗-product on an arbitrary symplectic manifold, we need a local form of this multiplication. However, a universal method of calculating the ${*}_{P}$-multiplication in that manner does not exist. However, if we substitute convergent expressions by their series in expansions in the Planck constant ℏ, we are able to propose a calculus called deformation quantisation, which is, in some cases, equivalent to the aforementioned considerations. The price we pay for this operation is a general loss of convergence.

Thus, we introduce a vector space $\left(C^{\infty }\left[\hbar^{-1},\hbar \right]\right]\left(R^{2}\right),C\left[\hbar^{-1},\hbar \right]],+,\cdot )$ of formal series in *ℏ* over the field of complex numbers extended with respect to parameter ℏ. Elements of the field $C[\hbar^{-1},\hbar ]]$ are of the form

$$\sum_{l=-r}^{\infty }\hbar^{l}c_{l},\;\;\;r\in N,\;\;\;\forall \;l\;c_{l}\in C$$

and the formal series belonging to $C^{\infty }\left[\hbar^{-1},\hbar \right]]\left(R^{2}\right)$ can be written as

(34) $$A\left(p,q\right)=\sum_{l=-s}^{\infty }\hbar^{l}A_{l}\left(p,q\right),\;\;\;s\in N,\;\;\;\forall \;l\;A_{l}\left(p,q\right)\in C^{\infty }\left(R^{2}\right).$$

A detailed analysis of the structure of the vector space $\left(C^{\infty }\left[\hbar^{-1},\hbar \right]\right]\left(R^{2}\right),C\left[\hbar^{-1},\hbar \right]],+,\cdot )$ is presented in [54]. To introduce a ${*}_{P}$-product of the formal series from $C^{\infty }\left[\hbar^{-1},\hbar \right]]\left(R^{2}\right)$, first, we build another formal series

(35) $$P\left(\hbar \lambda \mu \right)=\sum_{j=0}^{\infty }P_{j}\cdot {\left(\hbar \lambda \mu \right)}^{j},\;\;\;P_{0}=1,\;\;\forall \;j\rangle 0\;\;P_{j}\in R.$$

The formal series P(ℏλμ) is responsible for the choice of ordering. We will discuss this question in Section 5. As one can see, the series plays the same role as function P(ℏλμ) in the former considerations. Thus, we denote both of them with the same symbol.

Element $A_{P}\left(p,q\right)$, constructed from the formal series (34), is equal to

(36) $$A_{P}\left(p,q\right):=P\left(-\hbar \frac{\partial^{2}}{\partial p\partial q}\right)A\left(p,q\right)\overset{\left(34\right)}{=}\sum_{j=0}^{\infty }\sum_{l=-s}^{\infty }{\left(-1\right)}^{j}\hbar^{j+l}P_{j}\frac{\partial^{2j}\;A_{l}\left(p,q\right)}{\partial p^{\;j}\partial q^{\;j}}.$$

Multiplication of the formal series is then defined as

(37) $$A\left(p,q\right){*}_{P}B\left(p,q\right):=P^{-1}\left(-\hbar \frac{\partial^{2}}{\partial p\partial q}\right)\left[A_{P}\left(p,q\right)\exp\left(\frac{i\hbar \;\overset{↔}{P}}{2}\right)B_{P}\left(p,q\right)\right]$$

with the Poisson operator being given by the expression

$$\overset{↔}{P}:=\frac{\overset{\leftarrow }{\partial }}{\partial q}\frac{\vec{\partial }}{\partial p}-\frac{\overset{\leftarrow }{\partial }}{\partial p}\frac{\vec{\partial }}{\partial q}.$$

Function ’exp’ in formula (37) needs to be represented by its Taylor series.

The algebra $\left(C^{\infty }\left[\hbar^{-1},\hbar \right]\right]\left(R^{2}\right),C\left[\hbar^{-1},\hbar \right]],+,{*}_{P})$ can be equipped in a natural way with a Lie structure. The Moyal bracket (33) in set $\left(C^{\infty }\left[\hbar^{-1},\hbar \right]\right]\left(R^{2}\right),C\left[\hbar^{-1},\hbar \right]],+,{*}_{P})$ is equal to

(38) $${\left\{A\left(p,q\right),B\left(p,q\right)\right\}}_{M}=\frac{2}{\hbar }P^{-1}\left(-\hbar \frac{\partial^{2}}{\partial p\partial q}\right)\left[A_{P}\left(p,q\right)\sin\left(\frac{\hbar \;\overset{↔}{P}}{2}\right)B_{P}\left(p,q\right)\right].$$

Although we stress that the formal series approach to algebra of functions on the symplectic manifold $R^{2}$ may destroy convergence, it also possesses some advantages compared to the strict star calculus. First, it can be extended to all formal series of smooth functions on $R^{2}.$ Secondly, calculations involving exclusively derivatives become easier compared to integral expressions. Finally, for a wide class of objects, e.g., polynomials, the results of ${*}_{P}$-multiplication achieved with the use of expression (37) and (30) are the same.

As an example of the aforementioned product, we calculate

$$p{*}_{P}q=pq-\frac{i\hbar }{2}+\hbar P_{1}.$$

The formal series version of phase space quantum mechanics is our chance to move to nontrivial symplectic manifolds. Product (37) of the formal series is calculated locally. A natural way to transform it into a form suitable to work on arbitrary symplectic manifold appears to be the substitution of partial derivatives with covariant ones. This operation ensures that the result of the multiplication would be again a scalar.

Let us discuss this option in the simplest case when kernel P(ℏλμ)=1. The phase space is a symplectic manifold M,dimM=2n. Assuming

$$\frac{\partial A(\phi_{1},\ldots ,\phi_{2n})}{\partial \hbar }=\frac{\partial B(\phi_{1},\ldots ,\phi_{2n})}{\partial \hbar }=0$$

in a local chart $\left(U,\left\{\phi_{1},\ldots ,\phi_{2n}\right\}\right)$ (not necessarily a Darboux one) on manifold M, the term standing at power $\hbar^{n}$ in expression (37) is proportional to

(39) $$\omega^{i_{1}j_{1}}\ldots \omega^{i_{n}j_{n}}\nabla_{i_{1}}\cdots \nabla_{i_{n}}A\left(\phi_{1},\ldots \phi_{2n}\right)\nabla_{j_{1}}\cdots \nabla_{j_{n}}B\left(\phi_{1},\ldots \phi_{2n}\right).$$

By $\omega^{i_{k}j_{k}}$, we denote components of the tensor inverse to the symplectic form ω. In the chart $\left(U,\left\{\phi_{1},\ldots ,\phi_{2n}\right\}\right)$, represented by its components $\omega_{ij}$, $\nabla_{i}$, symbolises the covariant derivative with respect to the variable $\phi_{i}.$ The Einstein summation convention is used. The presented idea requires the existence of a connection on the phase space M.

Unfortunately, as is known (e.g., [20]), the ∗-product consisting of terms, like (39), is associative if and only if the connection on M is

1. symplectic $\nabla_{i}\omega =0\;\;\mathrm{for}\;\;i=1,2,\ldots ,2n;$
2. without torsion
  $$\nabla_{ij}A\left(\phi_{1},\ldots \phi_{2n}\right)=\nabla_{ji}A\left(\phi_{1},\ldots \phi_{2n}\right)\;\mathrm{for}\;i,j=1,2,\ldots ,2n\;\;$$
  and every function $A(\phi_{1},\ldots ,\phi_{2n})$; and
3. flat
  $$\nabla_{ijk}A\left(\phi_{1},\ldots \phi_{2n}\right)=\nabla_{ikj}A\left(\phi_{1},\ldots \phi_{2n}\right)\;\mathrm{for}\;i,j,k=1,2,\ldots ,2n\;$$
  and every function $A(\phi_{1},\ldots ,\phi_{2n})$.

These three constraints taken together are demanding. Despite the fact that every symplectic manifold can be equipped with several symplectic connections, there are usually some restrictions imposed on them. Indeed, most phase spaces are cotangent bundles with the base spaces being configuration spaces. Those configuration spaces usually contain a Riemannian connection. It is natural that the symplectic connection on $M=T^{*}V$ should be compatible with the Riemannian connection on the base manifold V. Although ‘compatibility’ can be realised in several ways (e.g., [55,56,57,58]), the final symplectic torsion-free connection may still be curved. Thus, the programme of generalisation of product (37) in the sense presented above fails.

There exists a constructive iterative method of building a ∗-product on an arbitrary symplectic manifold. This method was proposed by B. Fedosov in his outstanding works [59,60]. We are not able to present the whole algorithm invented by Fedosov; however, to give an idea, we will indicate its main steps.

The stage for the Fedosov calculus is a generalised Weyl bundle. The base manifold of that bundle is the symplectic manifold M at which we want to define the ∗-product. The fibre is an algebra of a formal series in *ℏ* of symmetric powers of the cotangent bundle $T^{*}M.$ Therefore, for a fixed chart on the U⊂M, elements of the fibre are formal series of polynomials of coordinates in $T^{*}M.$ The product ‘∘’ in the fibre of the generalised Weyl bundle is given by a formula similar to (37).

In the next step, the generalised Weyl algebra bundle is equipped with a symplectic torsion-free connection induced by the symplectic torsion-free connection from the base space M. Thus, phase space quantum mechanics, in contrast to classical Hamilton physics, require some connection. This connection on the Weyl bundle is then modified to an Abelian connection.

Finally, the formal series on the manifold M to be multiplied in the sense of star product is lifted to a flat section of the Weyl algebra bundle, then their ∘-product is calculated, and the result is projected on M.

A great advantage of the Fedosov construction is its recurrent character. Therefore, it can be easily implemented in the form of a computer programme [61,62]. However, we have to remember that this calculus is formal, and some formulas can be divergent.

The Fedosov method, apart from its unquestionable applicable virtue, answers a fundamental theoretical question. It shows that, on any symplectic manifold, one can introduce a nontrivial deformation of the Poisson structure. That fact was proven first by De Wilde and Lecomte [63]. Kontsevich [64] solved the problem of the existence of such a deformation of Poisson algebra for any Poisson manifold.

Let us analyse some properties of the ∗-product on an arbitrary symplectic manifold M. These properties can be deduced, e.g., from the Fedosov construction; however, here we propose another, axiomatic, point of view. We consider any mapping fulfilling these conditions as ∗-multiplication on M equivalent to the product of linear operators acting in the Hilbert space H. Thus, we look at the phase space representation of quantum mechanics from another perspective. If we are able to introduce a mapping ‘∗’ satisfying the axioms written below, we have a basis to construct quantum physics.

We postulate a ∗-product to be a two-argument multiplication in the linear space of the formal series $C^{\infty }\left[\hbar^{-1},\hbar \right]]\left(M\right)$ on a manifold M mapping every pair of formal series into another formal series

$$*:C^{\infty }\left[\hbar^{-1},\hbar \right]]\left(M\right)\times C^{\infty }\left[\hbar^{-1},\hbar \right]]\left(M\right)\to C^{\infty }\left[\hbar^{-1},\hbar \right]]\left(M\right).$$

1. For the functions $A\left(\phi_{1},\ldots ,\phi_{2n}\right)\;\;\mathrm{and}\;\;D\left(\phi_{1},\ldots ,\phi_{2n}\right)\;\in C^{\infty }\left(M\right)$, i.e., not containing the deformation parameter *ℏ*, we write the ∗-product in the form
  (40) $$A*D:=\sum_{k=0}^{\infty }\hbar^{k}B_{k}\left(A,D\right),\;\;\forall \;k\;\;B_{k}\left(A,D\right)\in C^{\infty }\left(M\right).$$
  To shorten the notation, we do not write the local coordinates in the arguments of functions.
2. The operators $B_{k}\left(\cdot ,\cdot \right),\;\;k=0,1,2,\ldots$ are $C[\lambda^{-1},\lambda ]]$–bilinear.
3. Moreover, they are local, i.e.,
  $$\forall \;A,D\in C^{\infty }\left(M\right)\;\;\;\mathrm{supp}\;B_{k}\left(A,D\right)\subset \left(\mathrm{supp}\;A\;\cap \mathrm{supp}\;D\right).$$
4. The ∗-product is associative. Thus, for every k≥0 and every functions $A,D,G\in C^{\infty }\left(M\right)$,
  $$\sum_{l=0}^{k}B_{l}\left(A,B_{k-l}\left(D,G\right)\right)=\sum_{l=0}^{k}B_{l}\left(B_{k-l}\left(A,D\right),G\right).$$
5. Next, for every $A,D\in C^{\infty }\left(M\right),$ the equality holds
  $$B_{0}\left(A,D\right)=A\cdot D.$$
  This requirement physically means that the ∗-product is, indeed, a deformation of the pointwise (classical) multiplication of functions.
6. For every k≥1 and every function $A\in C^{\infty }\left(M\right),$ there is
  $$B_{k}\left(1,A\right)=B_{k}\left(A,1\right)=0.$$
  Therefore, the constant function equal to 1 is the identity element with respect to the ∗-product.
7. For all $A,D\in C^{\infty }\left(M\right),$
  $$B_{1}\left(A,D\right)-B_{1}\left(D,A\right)=i{\left\{A,D\right\}}_{P},$$
  This condition ensures that, in the first approximation with respect to the deformation parameter *ℏ*, the difference
  $$\left(A*D-D*A\right)∼i\hbar {\left\{A,D\right\}}_{P}.$$
  The aforementioned relation explains why the ∗-multiplication leads to a deformation of the Poisson structure.
8. The complex conjugation is an involution of the ∗–algebra, i.e., for every $A,D\in C^{\infty }\left(M\right),$
  $$\bar{B_{k}\left(A,D\right)}=B_{k}\left(\bar{D},\bar{A}\right).$$
  Thus, we say that the ∗-product is Hermitian.
  There are two more supplementary assumptions about the ∗-product that are widely used. The first says that
9. The operators $B_{k}\left(\cdot ,\cdot \right),\;\;k=0,1,2,\ldots$ are bidifferential. This condition assures a convenient realisation of the previous requirements.
10. The second states that operator $B_{k}\left(\cdot ,\cdot \right),\;\;k=0,1,2,\ldots$ is, at most, of the order k. In this case, we call the ∗-product natural.

The set of series $C^{\infty }\left[\hbar^{-1},\hbar \right]]\left(M\right)$ with the ∗-product is a ring, which, in general, is nonabelian. Moreover, it is an algebra over the field $C[\hbar^{-1},\hbar ]].$ The ∗-product is continuous in its arguments if all of the operators $B_{k}\left(\cdot ,\cdot \right)$ are continuous. Thus, every natural ∗-multiplication is continuous.

The discussed construction and the properties of ∗-products work only on differentiable manifolds. Therefore, a phase space being a lattice requires a separate treatment. Thus, at the end of the current section, we are going to investigate the structure of set of observables on the (s+1)×(s+1) grid $\Gamma^{\left(s+1\right)}={\left\{\left(\varphi_{m},n\right)\right\}}_{m,n=0,\ldots ,s}$ being the phase space for the internal degrees of freedom, like spin.

We assume that observables are real functions on $\Gamma^{\left(s+1\right)}.$ They are elements of the vector space $\left(C\left(\Gamma^{\left(s+1\right)}\right),C,+,\cdot \right)$ of complex valued functions over the field of complex numbers C. Notions of continuity and smoothness are not defined there; however, we can introduce topology in the set $C(\Gamma^{\left(s+1\right)}).$ We say that a sequence of functions ${\left\{A_{j}\left(\phi_{m},n\right)\right\}}_{j=1}^{\infty }$ is convergent to a function $A(\phi_{m},n)$ if, for every fixed value of $\phi_{m_{0}}$ and $n_{0}$, the sequence of numbers ${\left\{A_{j}\left(\varphi_{m_{0}},n_{0}\right)\right\}}_{j=1}^{\infty }$ tends to the number $A(\phi_{m_{0}},n_{0}).$

Our goal is to equip the set of functions $C(\Gamma^{\left(s+1\right)})$ with a structure of noncommutative but associative algebra. The multiplication should be a counterpart of the product of linear operators acting in the finite dimensional Hilbert space $H^{\left(s+1\right)}.$ Applying the discrete Weyl correspondence (23), we obtain that the product of two functions $A\left(\phi_{m},n\right),\;B\left(\phi_{m},n\right)\in C\left(\Gamma^{\left(s+1\right)}\right)$ related to the operators $\hat{A},\;\hat{B}$ is determined by

(41) $$\left(A{*}_{K}B\right)\left(\phi_{m},n\right)=\frac{1}{s+1}\sum_{k,l=0}^{s}K^{-1}\left(k,l\right)\exp\left\{i\left(k\phi_{m}+\frac{2\pi }{s+1}ln\right)\right\}\times \mathrm{Tr}\left\{\hat{A}\cdot \hat{B}\;\;{\hat{D}}^{†}\left(k,l\right)\right\}.$$

To obtain the product (41) in a form not referring to operators, we need to introduce the auxiliary functions $\tilde{\tilde{A}}\left(k,l\right)$ and $\tilde{\tilde{B}}\left(k,l\right)$, defined on pairs of natural numbers {0,…,s}×{0,…,s} and calculated according to the rule

(42) $$\tilde{\tilde{A}}\left(k,l\right):=\frac{1}{{\left(s+1\right)}^{2}}K\left(k,l\right)\sum_{m,n=0}^{s}\exp\left\{\frac{-2\pi i(k\phi_{m}+n\phi_{l})}{s+1}\right\}A\left(\phi_{m},n\right).$$

Now, Equation (41) turns into

$$\left(A{*}_{K}B\right)\left(\varphi_{m},n\right)=\sum_{k,l=0}^{s}K^{-1}\left(k,l\right)\exp\left\{i\left(k\phi_{m}+\frac{2\pi }{s+1}ln\right)\right\}$$

$$\times \sum_{k^{\prime },k^{\prime \prime },l^{\prime },l^{\prime \prime }=0}^{s}\tilde{\tilde{A}}\left(k^{\prime },l^{\prime }\right){\left(-1\right)}^{\left(k^{\prime }-k\right)Y\left(l^{\prime }-l-1\right)+\left(l^{\prime }-l\right)Y\left(k^{\prime }-k-1\right)+\left(s+1\right)Y\left(k^{\prime }-k-1\right)Y\left(l^{\prime }-l-1\right)}$$

(43) $$\times \exp\left\{\frac{i\pi }{s+1}\left(k^{\prime }l-kl^{\prime }\right)\right\}\delta_{k^{\prime }+k^{\prime \prime }-k,0\;\mathrm{mod}\left(s+1\right)}\delta_{l^{\prime }+l^{\prime \prime }-l,0\;\mathrm{mod}\left(s+1\right)}\tilde{\tilde{B}}\left(k^{\prime \prime },l^{\prime \prime }\right).$$

By Y(k),k∈Z, we denote the discrete Heaviside step function

$$Y\left(k\right):=\left\{\begin{array}{cc} 1, & k\geq 0,\\ 0, & k\langle 0. \end{array}\right.$$

The Planck constant *ℏ* does not appear in formula (43). The effect originates from the fact that the internal degrees of freedom do not refer to any classical quantities and, thus, operations on them are not a deformation of their classical counterparts.

Set $\left(C\left(\Gamma^{\left(s+1\right)}\right),+,{*}_{K}\right)$ is a ring with identity. Since this is also a vector space over C, we have built an associative noncommutative algebra containing quantum observables. It is also a Lie algebra with the Lie bracket given by the Moyal bracket

(44) $${\left\{A\left(\varphi_{m},n\right),B\left(\varphi_{m},n\right)\right\}}_{M}:=A\left(\varphi_{m},n\right){*}_{K}B\left(\varphi_{m},n\right)-B\left(\varphi_{m},n\right){*}_{K}A\left(\varphi_{m},n\right).$$

By combining the results for a symplectic manifold M and a grid $\Gamma^{\left(s+1\right)}$ one can derive a phase space algebra of the functions for systems described by both classical and internal degrees of freedom.

## 5. Physically Motivated Orderings

As it was explained in the preceding chapter, in both continuous and discrete cases, we were able to construct several quantum algebras parametrised by functions: P(λ,μ) or K(k,l), which are often called kernels. Before we start discussion of role of them in the phase space version of quantum mechanics, we need to make the following comment.

Mathematicians divide the products of formal series into classes of equivalence. Thus, in deformation quantisation built on a symplectic manifold M, two products ${*}_{P_{1}}$ and ${*}_{P_{2}}$ are equivalent if there exists a differential operator

$$g:C^{\infty }\left(M\right)\to C^{\infty }\left(M\right)\;\;,\;\;g=1+\sum_{i=1}^{\infty }g_{i}$$

such that

(45) $$g\left(A{*}_{P_{1}}D\right)=g\left(A\right){*}_{P_{2}}g\left(D\right).$$

Thus, we see that most of our earlier considerations were devoted to star products from the same class. However, the physical meaning of the equivalent ∗-products is different, because they lead, e.g., to different eigenvalues and eigenvectors. Thus, from the point of view of the experiment, we have to distinguish between them, and this is why it is worth discussing several kernels.

Let us do that. The first observation, following from the analysis of units of arguments says that P(λ,μ) and K(k,l) should be dimensionless. Since the parameters λ,μ appear in the Fourier transform (10), the argument of function P(λ,μ) is the product ℏλμ. If the phase space is $R^{2n},$ then the argument equals $\hbar \sum_{j=1}^{n}\lambda^{j}\mu_{j}.$

Moreover, comparing the expressions (16) and (81), we see that factor $\frac{1}{1+s}$ imitates $\frac{\hbar }{2\pi }.$ This is why the argument $\frac{\hbar \lambda \mu }{2}$ of function P turns into the argument $\frac{\pi kl}{1+s}$ of the kernel K, and we often write $P\left(\frac{\hbar \lambda \mu }{2}\right)$ and $K\left(\frac{\pi kl}{s+1}\right).$

The next restriction follows from the fact that, in several formulas, the inverse elements $P^{-1}\left(\frac{\hbar \lambda \mu }{2}\right)$ and $K^{-1}\left(\frac{\pi kl}{s+1}\right)$ appear. Therefore, an essential property of every kernel is its invertibility. In strict integral expressions, it is sufficient to assume that $P(\frac{\hbar \lambda \mu }{2})$ is different from 0 almost everywhere and that singularities do not lead to divergent integrals. For the formal series, we see that there must be $P_{0}\neq 0.$ In the case of a discrete phase space $\Gamma^{\left(s+1\right)}$, we simply demand that $K\left(\frac{\pi kl}{s+1}\right)\neq 0$ for every k,l.

The presence of the functions $P(\frac{\hbar \lambda \mu }{2})$ and $K\left(\frac{\pi kl}{s+1}\right)$ in our considerations originates from the noncommutativity of the operators $\hat{p},\hat{q}$ on a continuous phase space $R^{2}$ and of the operators $\hat{n},\hat{\phi }$ on the grid $\Gamma^{\left(s+1\right)}$. Thus, it is natural that kernels should not interfere in systems depending exclusively on the momentum or the position and, analogously, exclusively on *n* or $\phi_{m}.$ This expectation means that P(0)=1 and K(0)=1. For the formal series $P(\frac{\hbar \lambda \mu }{2})$, the condition P(0)=1 assures the existence of the series $P^{-1}\left(\frac{\hbar \lambda \mu }{2}\right).$

Last, but not least, we expect that real functions correspond to the Hermitian operators. On symplectic manifolds, that requirement implies $P(\frac{\hbar \lambda \mu }{2})$ to be real. On the lattice $\Gamma^{\left(s+1\right)}$, the fact that real valued functions are mapped in Hermitian operators leads to the requirement

(46) $$\bar{K\left(\frac{\pi kl}{s+1}\right)}={\left(-1\right)}^{s+1-k-l}K\left(\frac{\pi (s+1-k)(s+1-l)}{s+1}\right),\;1\leq k,l\leq s,\;\;\bar{K(0)}=K\left(0\right).$$

Likely, the best known realisation of ordering on the phase space $R^{2}$ is the Weyl ordering. It holds for

$$P\left(\frac{\hbar \lambda \mu }{2}\right)=1.$$

One of the distinguishing features of the Weyl ordering is the fact that $P(\frac{\hbar \lambda \mu }{2})=1$ is the only kernel assuring compatibility for an arbitrary monomial $p^{r}q^{s},\;r,s\in N$ between the Poisson brackets and the respective commutators up to the second order, i.e.,

$$W_{1}\left({\left\{q^{2},p^{r}q^{s}\right\}}_{P}\right)=\frac{1}{i\hbar }\left[{\hat{q}}^{2},W_{1}\left(p^{r}q^{s}\right)\right]\;,\;W_{1}\left({\left\{p^{2},p^{r}q^{s}\right\}}_{P}\right)=\frac{1}{i\hbar }\left[{\hat{p}}^{2},W_{1}\left(p^{r}q^{s}\right)\right]$$

and

$$W_{1}\left({\left\{pq,p^{r}q^{s}\right\}}_{P}\right)=\frac{1}{i\hbar }\left[W_{1}\left(pq\right),W_{1}\left(p^{r}q^{s}\right)\right].$$

What is amazing, because of the requirement (46) in the discrete case, one cannot put $K\left(\frac{\pi kl}{s+1}\right)=1$ for every 1≤k,l≤s. A possible choice for s+1=oddnumber, is

$$K\left(\frac{\pi kl}{s+1}\right)={\left(-1\right)}^{kl}.$$

If s+1 is an even number, the situation becomes more complicated. A partial answer to the question about a possible kernel on a grid of even dimensions can be found in [48]. However, one can always choose $K\left(\frac{\pi kl}{s+1}\right)$, which is real and $\left|K\left(\frac{\pi kl}{s+1}\right)\right|=1.$

Another degree of freedom with respect to a choice of ordering appears in the Fedosov quantisation. As we mentioned, in the Fedosov construction, a symplectic torsion-free connection on the phase space M is required, and a given phase space may be equipped with different symplectic connections. Thus, we may build several ∗-products changing the differential structure of the manifold M. Moreover, every fibre in the Weyl bundle admits several ∘-products leading also to different ∗-products. Considerations devoted to this aspect of phase space quantum mechanics can be found in [58,65].

## 6. Representation of States on a Quantum Phase Space

The question of representing states on a quantum phase space should be discussed in three aspects. First, we need a phase space counterpart of a density operator. In the second step, a functional action equivalent to a trace in the Hilbert space has to be introduced. These two structures plus a ∗-product are sufficient to deal with calculating the mean values and the time evolution.

However, in quantum mechanics, we are also interested in the possible results of a single measurement. To gain this information, we solve an eigenvalue equation for a fixed observable. Thus, a phase space analogue is required.

As we said before, the density operator $\hat{\varrho }$ acting in the Hilbert space H is a linear, self-adjoint, positive, and normalised operator defined on the whole Hilbert space.

Since the density operator is self-adjoint and its domain is the whole space H, then it is bounded. Eigenvalues of the density operator are nonnegative, and they do not exceed 1. Thus, its norm satisfies the inequality $\left|\right|\hat{\varrho }\left|\right|\leq 1.$

The interpretation of the eigenvalues and eigenstates of this operator is clearly expressed in the von Neumann definition [66] stating that the density operator is of the form

$$\hat{\varrho }:=u\;-\;\lim_{n\to \dim H}\sum_{j=1}^{n}p_{j}\left|\varphi_{j}\rangle \langle \varphi_{j}\right|,\;\;\forall \;j\;\;p_{j}\geq 0\;\;,\;\;\sum_{j=1}^{\dim H}p_{j}=1.$$

Each number $p_{j},\;j=1,2,\ldots ,\dim H$ is the probability of observing the system in the state represented by a ket $|\varphi_{j}\rangle .$ If one of these numbers equals 1, we say that the system is in a pure state. Otherwise, the system is in a mixed state. The symbol u− denotes the uniform convergence of a sequence of operators.

Thus, we see that every pure state is represented by an operator of the projection on a 1-D subspace of the Hilbert space H.

The functional action of the density operator $\hat{\varrho }$ on an observable represented by the operator $\hat{A}$ is calculated with the use of trace. For any bounded and positive operator $\hat{A}$, its trace is defined as [67]

(47) $$\mathrm{Tr}\hat{A}:=\sum_{i=1}^{\dim H}\langle \varphi_{i}\left|\hat{A}\right|\varphi_{i}\rangle ,$$

where ${\left\{|\varphi_{i}\rangle \right\}}_{i=1}^{\dim H}$ constitutes a basis of the Hilbert space H. The trace (47) is independent of the basis chosen. Because of the equalities

$$\mathrm{Tr}\left(\hat{A}+\hat{B}\right)=\mathrm{Tr}\;\hat{A}+\mathrm{Tr}\;\hat{B}$$

$$\forall \;b\in C\;\;\mathrm{Tr}\left(b\hat{A}\right)=b\cdot \mathrm{Tr}\hat{A},$$

we see that the trace actually realises a linear functional. The trace is invariant under any unitary transformation.

For every density operator $\hat{\varrho }$,

$$\mathrm{Tr}\sqrt{{\hat{\varrho }}^{+}\hat{\varrho }}=\mathrm{Tr}\left|\hat{\varrho }\right|=\mathrm{Tr}\hat{\varrho }=1.$$

Thus, the density operator is a trace class operator. Therefore, for every bounded operator $\hat{A}\in B\left(H\right)$, the trace of the product $\hat{\varrho }\hat{A}$ is abelian

$$\mathrm{Tr}\left(\hat{\varrho }\hat{A}\right)=\mathrm{Tr}\left(\hat{A}\hat{\varrho }\right).$$

Since states are represented by density operators, we have to redefine the eigenvalue equation. Instead of formula

(48) $$\hat{A}\left|\varphi_{i}\rangle =a_{i}\right|\varphi_{i}\rangle ,$$

where $a_{i}$ denotes an eigenvalue assigned to eigenvector $|\varphi_{i}\rangle$, we look for an equation determining the projection operator $\left|\varphi_{i}\rangle \langle \varphi_{i}\right|$ as the density operator for the state $|\varphi_{i}\rangle .$ If the state is degenerated, we focus on one fixed vector $|\varphi_{i}\rangle$ from the subspace related to the eigenvalue $a_{i}.$

Thus, the eigenvalue equation (48) turns into

(49) $$\hat{A}{\hat{\varrho }}_{i}=a_{i}{\hat{\varrho }}_{i}.$$

However, there are several operators fulfilling (49) that are different from the projection operator $|\varphi_{i}\rangle \langle \varphi_{i}|.$ To extract the exact one referring to the state $|\varphi_{i}\rangle$, we need to use the following requirements:

1. The operator ${\hat{\varrho }}_{i}$ is self-adjoint, i.e., ${\hat{\varrho }}_{i}={\hat{\varrho }}_{i}^{†}.$
2. ${\hat{\varrho }}_{i}$ is an operator of the projection. Therefore, the equality ${\hat{\varrho }}_{i}={\hat{\varrho }}_{i}^{2}$ holds.
3. Its trace satisfies the formula $\mathrm{Tr}{\hat{\varrho }}_{i}=1.$

If the state referring to eigenvalue $a_{i}$ is degenerated, Equation (49) possesses more solutions satisfying the preceding conditions.

Density operators referring to different eigenvalues of the same self-adjoint operator satisfy the equality

$${\hat{\varrho }}_{i}{\hat{\varrho }}_{j}=\hat{0}.$$

There exist states that are not represented by density operators fulfilling the definitions presented at the beginning of this chapter, e.g., density operators assigned to the eigenstates of unbounded operators with continuous spectra. They usually do not fulfil the trace condition (3).

In the phase space formulation of quantum mechanics, a state given by the density operator $\hat{\varrho }$ is represented by an image of $\hat{\varrho }$ in the generalised Stratonovich–Weyl correspondence. For systems that are classically modelled on $R^{2}$, we know the explicit form of this mapping presented as the relation (13). By definition, an object

$$W_{P}\left(p,q\right):=W_{P}^{-1}\left(\frac{1}{2\pi \hbar }\hat{\varrho }\right)=\frac{1}{{\left(2\pi \right)}^{2}}\int_{R^{4}}P^{-1}\left(\frac{\hbar \lambda^{\prime }\mu^{\prime }}{2}\right)$$

(50) $$\times \exp\left(-\frac{i\hbar \lambda^{\prime }\mu^{\prime }}{2}\right)\exp\left[i\lambda^{\prime }\left(p-p^{\prime }\right)+i\mu^{\prime }\left(q-q^{\prime }\right)\right]\frac{\langle q^{\prime }\left|\hat{\varrho }\right|p^{\prime }\rangle }{{\left(2\pi \hbar \right)}^{1/2}}\exp\left(-\frac{ip^{\prime }q^{\prime }}{\hbar }\right)dp^{\prime }dq^{\prime }$$

is called a Wigner function. It depends on the choice of function $P\left(\frac{\hbar \lambda \mu }{2}\right).$ The role of factor $\frac{1}{2\pi \hbar }$ in front of the density operator $\hat{\varrho }$ will become clear later. Since the Wigner function is defined on the phase space and it represents the state of the system, it should be similar to the density of probability. This analogy is, however, misleading, because the function $W_{P}\left(p,q\right)$, although real, usually take both positive and negative values. Even at a spatial point from the interval $\left(q_{0},q_{1}\right)$, at which the density of the probability of detection of the system is 0, the Wigner function $W_{P}\left(p,q\right)$ for $q\in (q_{0},q_{1})$ may be different from 0 [68].

Information about probability in the Wigner function is contained in an indirect way. One can check that, for every physically motivated ordering $P\left(\frac{\hbar \lambda \mu }{2}\right)$,

(51) $$\int_{R}W_{P}\left(p,q\right)dp=\mathrm{Tr}\left(|q\rangle \langle q|\hat{\varrho }\right),$$

which is the probability of detecting the system under consideration at a spatial interval dq.

Analogously,

(52) $$\int_{R}W_{P}\left(p,q\right)dq=\mathrm{Tr}\left(|p\rangle \langle p|\hat{\varrho }\right)$$

represents the density of probability with respect to momentum p.

For a pure state given in the Hilbert space formulation by the density operator $\hat{\varrho }\;=\;|\psi \rangle \langle \psi |$, the respective Wigner function equals

$$W_{P}\left(p,q\right)=\frac{1}{{\left(2\pi \right)}^{2}}\int_{R^{4}}dp^{\prime }dq^{\prime }d\lambda^{\prime }d\mu^{\prime }\;P^{-1}\left(\frac{\hbar \lambda^{\prime }\mu^{\prime }}{2}\right)\exp\left(-\frac{i\hbar \lambda^{\prime }\mu^{\prime }}{2}\right)$$

(53) $$\times \exp\left[i\lambda^{\prime }\left(p-p^{\prime }\right)+i\mu^{\prime }\left(q-q^{\prime }\right)\right]\frac{\psi \left(q^{\prime }\right)\bar{\psi (p^{\prime })}}{{\left(2\pi \hbar \right)}^{1/2}}\exp\left(-\frac{ip^{\prime }q^{\prime }}{\hbar }\right).$$

For the Weyl ordering when $P\left(\frac{\hbar \lambda^{\prime }\mu^{\prime }}{2}\right)=1,$ one can calculate integrals with respect to $d\lambda^{\prime }$ and $d\mu^{\prime }.$ Then, the expression (54) reduces to (see [26]) a simple form,

$$W_{1}\left(p,q\right)=\frac{1}{2\pi \hbar }\int_{R}d\xi \exp\left(\frac{ip\xi }{\hbar }\right)\bar{\psi \left(q+\frac{\xi }{2}\right)}\psi \left(q-\frac{\xi }{2}\right).$$

This Wigner function $W_{P}\left(p,q\right)$ representing a pure state, fulfils the condition

(54) $$W_{P}\left(p,q\right){*}_{P}W_{P}\left(p,q\right)=\frac{1}{2\pi \hbar }W_{P}\left(p,q\right).$$

The relation (54) is a straightforward consequence of observation, in that the density operator representing a pure state is a projection operator $|\varphi_{i}\rangle \langle \varphi_{i}|.$

For two Wigner functions $W_{i\;P}\left(p,q\right),\;W_{j\;P}\left(p,q\right)$ representing mutually orthogonal states $|\psi_{i}\rangle$ and $|\psi_{j}\rangle$, their product

(55) $$W_{i\;P}\left(p,q\right){*}_{P}W_{j\;P}\left(p,q\right)=0.$$

Thus, from the von Neumann definition of a density operator, we see that, if the Wigner functions $W_{i\;P}\left(p,q\right),\;i=1,2,\ldots$ represent projections on mutually orthogonal states spanning the Hilbert space H, then

(56) $$W_{P}\left(p,q\right)=\sum_{i=1}^{\dim H}p_{i}W_{i\;P}\left(p,q\right)$$

where $\forall \;i\;\;p_{i}\geq 0$ and $\sum_{i=1}^{\dim H}p_{i}=1.$

To prove the relations (54), (55), and (56), it is sufficient to assume the existence of the generalised Stratonovich–Weyl correspondence. Therefore, the aforementioned properties hold on any quantum phase space that is a symplectic manifold.

Another question is how the functional action of the Wigner function is realised. Using the generalised Weyl correspondence (13), we see that on the manifold $R^{2}$, the trace of the operator $\hat{A}$ is represented by the integral

(57) $$\frac{1}{2\pi \hbar }\int_{R^{2}}dpdqW_{P}^{-1}\left(\hat{A}\right).$$

This observation is not a surprise because integration, like taking the trace, introduces a linear functional. When one calculates the integral of a ${*}_{P}$-product of functions, the result of the integration is independent from the order of the multiplied functions

(58) $$\frac{1}{2\pi \hbar }\int_{R^{2}}dpdqA\left(p,q\right){*}_{P}B\left(p,q\right)=\frac{1}{2\pi \hbar }\int_{R^{2}}dpdqB\left(p,q\right){*}_{P}A\left(p,q\right).$$

Therefore, we conclude that, at phase space $R^{2}$, every ∗-product of (30) type is closed [69].

For the Weyl ordering, when $P\left(\frac{\hbar \lambda \mu }{2}\right)=1,$ the integral of the ${*}_{1}$-product of two functions is equal to the integral of the pointwise multiplication of those functions

(59) $$\frac{1}{2\pi \hbar }\int_{R^{2}}dpdqA\left(p,q\right){*}_{1}B\left(p,q\right)=\frac{1}{2\pi \hbar }\int_{R^{2}}dpdqA\left(p,q\right)\cdot B\left(p,q\right).$$

For an arbitrary ordering $P\left(\frac{\hbar \lambda \mu }{2}\right)$, the mean value of an observable A(p,q) in a state represented by a Wigner function $W_{P}\left(p,q\right)$ is calculated according to the rule

(60) $$\langle A(p,q)\rangle =\int_{R^{2}}dpdqA\left(p,q\right){*}_{P}W_{P}\left(p,q\right)=\int_{R^{2}}dpdqW_{P}\left(p,q\right){*}_{P}A\left(p,q\right).$$

Now, one can see that we have included the factor $\frac{1}{2\pi \hbar }$ into our definition of the Wigner function in order to avoid this coefficient in the expression for the average value.

The Weyl ordering formula (60) takes exactly the form known from classical statistical physics

(61) $$\langle A(p,q)\rangle =\int_{R^{2}}dpdqA\left(p,q\right)W_{1}\left(p,q\right).$$

The Wigner function $W_{1}\left(p,q\right),$ on the contrary to the classical density of probability, is not a positive function.

Once we introduce a classical counterpart of trace of operators, we can present more properties of the Wigner function. Thus, the integral

(62) $$\int_{R^{2}}dpdqW_{P}\left(p,q\right)=1.$$

Formula (62) is the condition of normalisation for the Wigner function $W_{P}\left(p,q\right).$ Moreover,

(63) $$\int_{R^{2}}dpdqW_{P}\left(p,q\right){*}_{P}W_{P}\left(p,q\right)\leq \frac{1}{2\pi \hbar }.$$

Equality in formula (63) holds exclusively for pure states.

Although a Wigner function is not positive as a function, it represents a positive functional. Thus, for every two Wigner functions $W_{a\;P}\left(p,q\right),\;W_{b\;P}\left(p,q\right)$,

(64) $$\int_{R^{2}}dpdqW_{a\;P}\left(p,q\right){*}_{P}W_{b\;P}\left(p,q\right)\geq 0.$$

Alternatively, one can say that, for every function A(q,p) and every Wigner function $W_{P}\left(p,q\right)$ for which the integral exists,

(65) $$\int_{R^{2}}dpdqW_{P}\left(p,q\right){*}_{P}\left(A\left(q,p\right){*}_{P}\bar{A(q,p)}\right)\geq 0.$$

The transition probability between two pure states represented by density matrices ${\hat{\varrho }}_{i}\;,\;{\hat{\varrho }}_{j}$ is given by integral

(66) $$|\langle \psi_{i}|\psi_{j}\rangle {|}^{2}=\mathrm{Tr}\left({\hat{\varrho }}_{i}{\hat{\varrho }}_{j}\right)=2\pi \hbar \int_{R^{2}}dpdqW_{i\;P}\left(p,q\right){*}_{P}W_{j\;P}\left(p,q\right).$$

Wigner functions $W_{i\;P}\left(p,q\right)\;,\;W_{j\;P}\left(p,q\right)$ refer to the pure states $|\psi_{i}\rangle ,\;|\psi_{j}\rangle$, respectively.

In the case where the phase space is a symplectic manifold M, but an explicit form of the generalised Stratonovich–Weyl correspondence is not known, we ought to understand a Wigner function as a phase space counterpart of the density operator $\hat{\varrho }.$ Thus, W is a real, positively defined, and normalised generalised function. Below, we present a realisation of these postulates.

1. For every real, smooth function $A(\phi_{1},\ldots ,\phi_{2n})$ from a proper test class of functions
  (67) $${\langle W\left(\phi_{1},\ldots ,\phi_{2n}\right),A\left(\phi_{1},\ldots ,\phi_{2n}\right)\rangle }_{*}\in R.$$
  The choice of class of test function may be performed according to several criteria. We discussed this question in [54]. By $\phi_{1},\ldots ,\phi_{2n}$, we denote the local coordinates (not necessarily canonical) on the manifold $M,\;n=\frac{1}{2}\dim M.$ The symbol ${\langle \cdot ,\cdot \rangle }_{*}$ represents a functional linear action. Its possible form will be discussed later. We omit the index P at the star product, because, as we know, on an arbitrary symplectic manifold depends not only on a choice of ordering but also on the geometry of the symplectic space.
2. For every test function,
  (68) $${\langle W\left(\phi_{1},\ldots ,\phi_{2n}\right),A\left(\phi_{1},\ldots ,\phi_{2n}\right)*\bar{A(\phi_{1},\ldots ,\phi_{2n})}\rangle }_{*}\geq 0.$$
3. The functional action on a constant function
  (69) $${\langle W(\phi_{1},\ldots ,\phi_{2n}),1\rangle }_{*}=1.$$

In the practical realisation of the preceding postulates, one has to overcome two serious obstacles—the introduction a ∗-product on a symplectic manifold M and the implementation of the functional action ${\langle \cdot ,\cdot \rangle }_{*}.$ As we remember from Section 4, a star product on an arbitrary symplectic manifold can be calculated in frames of the formal series calculus. Thus, the expressions (67), (68), and (69) need to be adapted to that calculus (compare [54]). In particular, we have to be extremely careful in dealing with Wigner functions. Analysis of the solutions of eigenvalue equations in that situation show [70] that Wigner functions may be represented as a formal series in ℏ, in which the terms are the Dirac delta and its derivatives. From that point of view, e.g., the sense of properties (54) and (55) is obscure.

Another question, intimately related to the definition of the functional action ${\langle \cdot ,\cdot \rangle }_{*},$ is a phase space representation of a trace of operators. Due to the linearity, it has to be, up to a constant, a kind of integral on the symplectic manifold M. However, the most natural integral

$$\int_{M}A\left(\phi_{1},\ldots ,\phi_{2n}\right)\omega^{n}$$

does not ensure that

$$\int_{M}A\left(\phi_{1},\ldots ,\phi_{2n}\right)*B\left(\phi_{1},\ldots ,\phi_{2n}\right)\omega^{n}=\int_{M}B\left(\phi_{1},\ldots ,\phi_{2n}\right)*A\left(\phi_{1},\ldots ,\phi_{2n}\right)\omega^{n}$$

for the arbitrary functions $A\left(\phi_{1},\ldots ,\phi_{2n}\right),B\left(\phi_{1},\ldots ,\phi_{2n}\right),$ for which integrals make sense. Therefore, it is necessary to introduce the so-called trace density. The existence of the trace density was proven in [71,72]. Its construction for the Weyl-like Fedosov star product was proposed by Boris Fedosov himself [73]. The trace density denoted as $t(\phi_{1},\ldots ,\phi_{2n})$ is a formal series in nonnegative powers of *ℏ* and was determined by a symplectic curvature tensor on the manifold M and its covariant derivatives with respect to a symplectic connection on M. Now,

$$\int_{M}\left(A\left(\phi_{1},\ldots ,\phi_{2n}\right)*B\left(\phi_{1},\ldots ,\phi_{2n}\right)\right)t\left(\phi_{1},\ldots ,\phi_{2n}\right)\omega^{n}$$

(70) $$=\int_{M}\left(B\left(\phi_{1},\ldots ,\phi_{2n}\right)*A\left(\phi_{1},\ldots ,\phi_{2n}\right)\right)t\left(\phi_{1},\ldots ,\phi_{2n}\right)\omega^{n}$$

as expected.

Now, we see that the functional action ${\langle f\left(\phi_{1},\ldots ,\phi_{2n}\right),A\left(\phi_{1},\ldots ,\phi_{2n}\right)\rangle }_{*}$ is a generalisation of the integral

(71) $$\frac{1}{{\left(2\pi \hbar \right)}^{n}}\int_{M}f\left(\phi_{1},\ldots ,\phi_{2n}\right)*A\left(\phi_{1},\ldots ,\phi_{2n}\right)t\left(\phi_{1},\ldots ,\phi_{2n}\right)\omega^{n}.$$

Factor $\frac{1}{{\left(2\pi \hbar \right)}^{n}}$ appears to assure compatibility with the case of $R^{2n}.$ Its origin is a relation between the trace of operators and the integration on the quantum phase space.

Therefore, for an observable $A(\phi_{1},\ldots ,\phi_{2n}),$ its average value is determined by the relation

(72) $$\langle A(\phi_{1},\ldots ,\phi_{2n})\rangle =\int_{M}\left(A\left(p_{1},\ldots ,q^{n}\right)*W\left(\phi_{1},\ldots ,\phi_{2n}\right)\right)t\left(\phi_{1},\ldots ,\phi_{2n}\right)\omega^{n}.$$

The scheme of dealing with the states described above is based on an assumption that the Wigner function represents the density operator in the Stratonovich–Weyl correspondence. This can be also applied to discrete problems.

However, on the phase space $R^{2n}$ and on the discrete phase space, the presented way of building the Wigner function is not unique. As an illustration of an alternative approach, we construct a Wigner function for the nonclassical degrees of freedom on a grid $\Gamma^{\left(s+1\right)}$.

We introduce the discrete Wigner function as

(73) $$W_{K}\left(\varphi_{m},n\right):=\mathrm{Tr}\left(\frac{1}{s+1}\hat{\varrho }\;\hat{\Omega }\left[K\right]\left(\varphi_{m},n\right)\right).$$

Thus,

(74) $$\langle A(\varphi_{m},n)\rangle =\sum_{m,n=0}^{s}A\left(\varphi_{m},n\right)\cdot W_{K}\left(\varphi_{m},n\right).$$

In contrast to the Wigner function based on the Stratonovich–Weyl correspondence, in the expression for the mean value of the function $A(\varphi_{m},n)$, we apply the usual pointwise multiplication between $A(\varphi_{m},n)$ and $W_{K}\left(\varphi_{m},n\right).$

The Wigner function defined by (73) satisfies the following properties:

1. It is a real function
  (75) $$W_{K}\left(\varphi_{m},n\right)=\bar{W_{K}\left(\varphi_{m},n\right)}.$$
2. The trace of $W_{K}\left(\varphi_{m},n\right)$ is equal to one
  (76) $$\sum_{m,n=0}^{s}W_{K}\left(\varphi_{m},n\right)=1.$$
  Thus, we see that the Wigner function is normalised as expected.
3. $W_{K}\left(\varphi_{m},n\right)$ is positively defined, i.e., for every function $A(\varphi_{m},n)$ on grid $\Gamma^{\left(s+1\right)}$,
  $$\sum_{m,n=0}^{s}W_{K}\left(\varphi_{m},n\right)\left(A{*}_{K}\bar{A}\right)\left(\varphi_{m},n\right)\geq 0.$$
4. It gives the marginal distributions
  (77) $$\sum_{m=0}^{s}W_{K}\left(\varphi_{m},n\right)=\mathrm{Tr}\{\hat{\rho }\;|n\rangle \langle n|\}$$
  (78) $$\sum_{n=0}^{s}W_{K}\left(\varphi_{m},n\right)=\mathrm{Tr}\{\hat{\rho }\;|\varphi_{m}\rangle \langle \varphi_{m}|\}.$$
  Thus, we can conclude that the Wigner function $W_{K}\left(\varphi_{m},n\right)$ has no direct probabilistic interpretation, but the sums in one of its arguments (77) or (78) are probabilities with respect to the fixed value *n* of the observable *n* or fixed value $\varphi_{m}$ for the function φ.

In both cases, the discrete and continuous, the crucial advantage of the Wigner function $W_{K}\left(\varphi_{m},n\right)$ over $W_{K}\left(\varphi_{m},n\right)$ and, respectively, $W_{P}\left(p,q\right)$ over $W_{P}\left(p,q\right)$ is that, in formulas for the average values of the functions $A\left(\varphi_{m},n\right),\;A\left(p,q\right)$, the ${*}_{K}$-multiplication or ${*}_{P}$-product, respectively, is substituted by the usual commutative product of functions. However, when one discusses an eigenvalue equation, the pure states, or the time evolution, it is more convenient to deal with Wigner functions obtained via the Stratonovich–Weyl correspondence. Therefore, in further considerations, we will use the Wigner functions $W_{P}\left(p,q\right),\;W_{K}\left(\varphi_{m},n\right)$ and $W(\phi_{1},\ldots ,\phi_{2n}).$

For $\left|P\left(\frac{\hbar \lambda \mu }{2}\right)\right|=1$, almost everywhere or $\left|K\left(\frac{\pi kl}{s+1}\right)\right|=1$, these two discussed approaches coincide.

We consider the representation of states in the case of the quantum phase space being a symplectic manifold and a grid separately. However, as it was shown in formula (27), there is a natural way to combine these two variants at least for the symplectic space $R^{2n}$ and an arbitrary grid $\Gamma^{\left(s+1\right)}.$ One needs only to treat the continuous and discrete components of the Wigner function on equal footing, i.e., to use the Wigner function derived via the Stratonovich–Weyl correspondence or via the trace.

The last paragraphs of the current section are devoted to issues in which the formulation is universal in all considered options: on the manifold $R^{2},$ on an arbitrary symplectic manifold M, on the grid $\Gamma^{\left(s+1\right)}$, and on the Cartesian products $R^{2}\times \Gamma^{\left(s+1\right)}$ or $M\times \Gamma^{\left(s+1\right)}.$

The time evolution of a Wigner function built with the use of the Stratonovich–Weyl correspondence or the generalised Stratonovich–Weyl correspondence is given by the Liouville–von Neumann–Wigner equation

(79) $$\frac{\partial W}{\partial t}+{\left\{W,H\right\}}_{M}=0.$$

This formula is a phase space counterpart of the time dependence for the density operator $\hat{\varrho }$ in the Schrödinger picture. By *H*, a Hamilton function is denoted. To avoid complicated notation, we omit the variables.

The eigenvalue equation for an observable *A*, on an arbitrary phase space takes the form

(80) $$A*W_{i}=a_{i}W_{i}.$$

The relation (80) refers to Wigner functions built with the use of the Stratonovich–Weyl correspondence or the generalised Stratonovich–Weyl correspondence. By $a_{i}$, an eigenvalue of quantity *A* is denoted. $W_{i}$ refers to a Wigner eigenfunction of *A* related with the eigenvalue $a_{i}.$ The conditions imposed on the Wigner function $W_{i}$ are the following. First, it is a real function. Secondly, as a counterpart of a projection operator, every Wigner eigenfunction satisfies the equality

(81) $$W_{i}*W_{i}=\frac{1}{{\left(2\pi \hbar \right)}^{n}\left(s+1\right)}W_{i}.$$

The coefficient $\frac{1}{{\left(2\pi \hbar \right)}^{n}\left(s+1\right)}$ follows from considerations related to the traces.

Finally, since $W_{i}$ represents a pure state, its action on a constant function equal to 1 everywhere gives 1. Symbolically,

(82) $$\sum_{m,l=0}^{s}\int_{M}W_{i}\left(\phi_{1},\ldots ,\phi_{2n},\varphi_{m},l\right)t\left(\phi_{1},\ldots ,\phi_{2n}\right)\omega^{n}=1.$$

Every Wigner eigenfunction $W_{i}$ commutes with the function *A*, i.e.,

(83) $${\left\{A,W_{i}\right\}}_{M}=0$$

their Moyal bracket vanishes.

A detailed analysis of the application of the Wigner function to represent the states of a spin $\frac{1}{2}$ nonrelativistic particle in a classical electromagnetic field can be seen in [49]. The quantum phase space in that example is $R^{3}\times R^{3}\times {\left\{\left(\phi_{m},n\right)\right\}}_{m,n=0,1}.$

## 7. Conclusions

The phase space version of quantum mechanics appears to be an interesting attempt to describe quantum phenomena. In several cases, we modelled on differentiable manifolds; therefore, we were able to apply the methods of differential geometry. Thus, the fact that the phase space is different from $R^{2n},$ is not a real obstacle.

However, there are also serious disadvantages of this formalism. The most acute ones follow from the fact that the quantum phase space may be different from the phase space of the classical counterpart of the system under consideration. We should also be aware of technical problems. Calculations performed in phase space quantum mechanics are typically very complicated. When formal series are in use, problems with the interpretation of the results appear.

It is also somewhat disappointing that the realisation of the classical limit in phase space quantum mechanics is not so straightforward. The naive definition of that limit, i.e., tending with the Planck constant to 0, works for the ∗-product and quantities containing positive powers of ℏ. However, the same simple mathematical operation for Wigner functions fails. Therefore, the phase space quantum mechanics cannot be treated directly as one of the approximated methods of the quantum formalism. An interpretation of the manipulations performed in phase space quantum mechanics require much deliberation.

## Data Availability

Not applicable.

## References

[1] Heisenberg W. (1985). Über quantentheoretische Umdeutung kinematischer und mechanischer Beziehungen. Original Scientific Papers Wissenschaftliche Originalarbeiten.

[2] Born M., Jordan P. (1925). Zur Quantenmechanik. I. Z. Phys..

[3] Born M., Heisenberg W., Jordan P. (1926). Zur Quantenmechanik. II. Z. Phys..

[4] Schrödinger E. (1926). Quantisierung als Eigenwertproblem. Ann. Phys. IV.

[5] Schrödinger E. (1926). Über das Verhältnis der Heisenberg Born Jordanischen Quantenmechanik zu der meinen. Ann. Phys. IV.

[6] Weyl H. (1927). Quantenmechanik und Gruppentheorie. Z. Phys..

[7] Weyl H. (1931). The Theory of Groups and Quantum Mechanics.

[8] Wigner E.P. (1932). On the Quantum Correction to Thermodynamic Equilibrium. Phys. Rev..

[9] Groenewold H.J. (1946). On the Principles of Elementary Quantum Mechanics. Physica.

[10] Moyal J.E. (1949). Quantum Mechanics as a Statistical Theory. Proc. Camb. Philos. Soc..

[11] Bartlett M.S., Moyal J.E. (1949). The Exact Transition Probabilities of Quantum–Mechanical Oscillators Calculated by the Phase–Space Method. Proc. Camb. Philos. Soc..

[12] Takabayasi T. (1954). The Formulation of Quantum Mechanics in Terms of Ensemble in Phase Space. Prog. Theor. Phys..

[13] Baker G. (1958). Formulation of Quantum Mechanics Based on the Quasi–Probability Distribution Induced on Phase Space. Phys. Rev..

[14] Fairlie D. (1964). The Formulation of Quantum Mechanics in Terms of Phase Space Functions. Proc. Camb. Philos. Soc..

[15] Plebański J.F. (1968). Poisson Brackets and Commutators.

[16] Agarwal G.S., Wolf E. (1970). Calculus for Functions of Noncommuting Operators and General Phase-Space Methods in Quantum Mechanics. I. Mapping Theorems and Ordering of Functions of Noncommuting Operators. Phys. Rev. D.

[17] Agarwal G.S., Wolf E. (1970). Calculus for Functions of Noncommuting Operators and General Phase-Space Methods in Quantum Mechanics. II. Quantum Mechanics in Phase Space. Phys. Rev. D.

[18] Agarwal G.S., Wolf E. (1970). Calculus for Functions of Noncommuting Operators and General Phase-Space Methods in Quantum Mechanics. III. A Generalized Wick Theorem and Multitime Mapping. Phys. Rev. D.

[19] Bayen F., Flato M., Fronsdal C., Lichnerowicz A., Sternheimer D. (1977). Quantum Mechanics as a Deformation of Classical Mechanics. Lett. Math. Phys..

[20] Bayen F., Flato M., Fronsdal C., Lichnerowicz A., Sternheimer D. (1978). Deformation Theory and Quantization: I. Deformations of Symplectic Structures. Ann. Phys..

[21] Bayen F., Flato M., Fronsdal C., Lichnerowicz A., Sternheimer D. (1978). Deformation Theory and Quantization: II. Physical Applications. Ann. Phys..

[22] Kim Y.S., Noz M.E. (1991). Phase Space Picture of Quantum Mechanics: Group Theoretical Approach.

[23] Schroeck F.E. (1994). Quantum Mechanics on Phase Space.

[24] Schleich W. (2001). Quantum Optics in Phase Space.

[25] Zachos C.K., Fairlie D.B., Curtright T.L. (2005). Quantum Mechanics in Phase Space.

[26] Tatarskii V.I. (1983). The Wigner Representation of Quantum Mechanics. Sov. Phys. Usp..

[27] Hillery M., O’Connell R.F., Scully M.O., Wigner E.P. (1984). Distribution Functions in Physics: Fundamentals. Phys. Rep..

[28] Lee H.W. (1995). Theory and Application of the Quantum Phase–Space Distribution Functions. Phys. Rep..

[29] Dito G., Sternheimer D. Deformation Quantization. Proceedings of the Meeting of Theoretical Physicists and Mathematicians.

[30] Waldmann S. (2007). Poisson–Geometrie und Deformationsquantisierung.

[31] Bengtsson I., Życzkowski K. (2006). Geometry of Quantum States.

[32] Mielnik B. (1968). Geometry of Quantum States. Commun. Math. Phys..

[33] Dirac P.A.M. (1925). The Fundamental Equations of Quantum Mechanics. Proc. R. Soc. Lond. Ser. A.

[34] Dirac P.A.M. (1930). The Principles of Quantum Mechanics.

[35] von Neumann J. (1932). Mathematische Grundlagen der Quantenmechanik.

[36] Bohm A. (1993). Quantum Mechanics: Foundations and Applications.

[37] Prugovečki E. (1981). Quantum Mechanics in Hilbert space.

[38] Bohm A., Gadella M. (1989). Dirac Kets, Gamov Vectors and Gel’fand Triplets.

[39] Thirring W. (1981). A Course in Mathematical Physics. III. Quantum Mechanics of Atoms and Molecules.

[40] Plebański J.F., Przanowski M., Tosiek J. (1996). The Weyl–Wigner–Moyal Formalism II. The Moyal Bracket. Acta Phys. Pol. B.

[41] Cariñena J.F., Gracia-Bondía J.M., Várilly J.C. (1990). Relativistic Quantum Kinematics in the Moyal Representation. J. Phys. A Math. Gen..

[42] Gadella M. (1995). Moyal Formulation of Quantum Mechanics. Fortschr. Phys..

[43] Plebański J.F., Przanowski M., Turrubiates F.J. (2000). Weyl-Underhill-Emmrich quantization and the Stratonovich-Weyl quantizer. J. Phys. A Math. Gen..

[44] Plebański J.F., Przanowski M., Tosiek J., Turrubiates F.J. (2000). Remarks on Deformation Quantization on the Cylinder. Acta Phys. Pol. B.

[45] Gonzalez J.A., del Olmo M.A., Tosiek J. (2003). Quantum Mechanics on the Cylinder. J. Opt. B.

[46] Przanowski M., Brzykcy P., Tosiek J. (2014). From the Weyl Quantization of a Particle on the Circle to Number–Phase Wigner Functions. Ann. Phys..

[47] Przanowski M., Brzykcy P., Tosiek J. (2015). Corrigendum to “From the Weyl Quantization of a Particle on the Circle to Number–Phase Wigner Functions” [Ann. Physics 351 (2014) 919–934]. Ann. Phys..

[48] Przanowski M., Tosiek J. (2017). From the Discrete Weyl–Wigner Formalism for Symmetric Ordering to a Number–Phase Wigner Function. J. Math. Phys..

[49] Przanowski M., Tosiek J., Turrubiates F.J. (2019). The Weyl–Wigner–Moyal Formalism on a Discrete Phase Space. I. A Wigner Function for a Nonrelativistic Particle with Spin. Fortschr. Phys..

[50] Gracia-Bondía J.M., Várilly J.C. (1988). Phase–Space Representation for Galilean Quantum Particles of Arbitrary Spin. J. Phys. A Math. Gen..

[51] Várilly J.C., Gracia-Bondía J.M. (1989). The Moyal Representation for Spin. Ann. Phys..

[52] Schwartz L. (1965). Méthodes Mathématiques pour les Sciences Physiques.

[53] Gracia-Bondía J.M., Várilly J.C. (1988). Algebras of Distributions Suitable for Phase–Space Quantum Mechanics. I. J. Math. Phys..

[54] Tosiek J., Dobrski M. (2019). Formal Series of Generalized Functions and Their Application to Deformation Quantization. J. Math. Phys..

[55] Bordemann M., Neumaier N., Waldmann S. (1998). Homogeneous Fedosov Star Products on Cotangent Bundles I: Weyl and Standard Ordering with Differential Operator Representation. Commun. Math. Phys..

[56] Bordemann M., Neumaier N., Waldmann S. (1999). Homogeneous Fedosov Star Products on Cotangent Bundles II: GNS Representations, the WKB Expansion, Traces, and Applications. J. Geom. Phys..

[57] Plebański J.F., Przanowski M., Turrubiates F.J. (2001). Induced Symplectic Connection on the Phase Space. Acta Phys. Pol. B.

[58] Tosiek J. (2011). Compatible Symplectic Connections on a Cotangent Bundle and the Fedosov Quantization. J. Math. Phys..

[59] Fedosov B. (1994). A Simple Geometrical Construction of Deformation Quantization. J. Differ. Geom..

[60] Fedosov B. (1996). Deformation Quantization and Index Theory.

[61] Tosiek J. (2008). The Fedosov ∗-Product in Mathematica. Comput. Phys. Commun..

[62] Tosiek J. (2010). The Fedosov ∗-Product in Mathematica. Comput. Phys. Commun..

[63] De Wilde M., Lecomte P. (1983). Existence of Star–Products and of Formal Deformations of the Poisson Lie Algebra of Arbitrary Symplectic Manifolds. Lett. Math. Phys..

[64] Kontsevich M. (2003). Deformation Quantization of Poisson Manifolds. Lett. Math. Phys..

[65] Przanowski M., Tosiek J. (1999). The Weyl–Wigner–Moyal Formalism. III. The Generalized Moyal Product in the Curved Phase Space. Acta Phys. Pol. B.

[66] von Neumann J. (1927). Wahrscheinlichkeitstheoretischer Aufbau der Quantenmechanik. Göttinger Nachr..

[67] Reed M., Simon B. (1972). Methods of Modern Mathematical Physics. I. Functional Analysis.

[68] Tosiek J., Cordero R., Turrubiates F.J. (2016). The Wentzel–Kramers–Brillouin Approximation Method Applied to the Wigner Function. J. Math. Phys..

[69] Connes A., Flato M., Sternheimer D. (1992). Closed Star Products and Cyclic Cohomology. Lett. Math. Phys..

[70] Dias N.C., Prata J.N. (2004). Formal Solutions of Stargenvalue Equations. Ann. Phys..

[71] Nest R., Tsygan B. (1995). Algebraic Index Theorem. Commun. Math. Phys..

[72] Gutt S., Rawnsley J. (2002). Traces for Star Products on Symplectic Manifolds. J. Geom. Phys..

[73] Fedosov B. (2002). Deformation quantization. Proceedings of the Meeting of Theoretical Physicists and Mathematicians (Strasbourg, France, 31 May–2 June, 2001).

